# Contact-electrification-activated artificial afferents at femtojoule energy

**DOI:** 10.1038/s41467-021-21890-1

**Published:** 2021-03-11

**Authors:** Jinran Yu, Guoyun Gao, Jinrong Huang, Xixi Yang, Jing Han, Huai Zhang, Youhui Chen, Chunlin Zhao, Qijun Sun, Zhong Lin Wang

**Affiliations:** 1 Beijing Institute of Nanoenergy and Nanosystems, Chinese Academy of Sciences, Beijing, China; 2 School of Nanoscience and Technology, University of Chinese Academy of Sciences, Beijing, China; 3 Center on Nanoenergy Research, School of Physical Science and Technology, Guangxi University, Nanning, China; 4 School of Materials Science and Engineering, Georgia Institute of Technology, Atlanta, GA United States

**Keywords:** Electrical and electronic engineering, Materials for devices, Nanoscale devices

## Abstract

Low power electronics endowed with artificial intelligence and biological afferent characters are beneficial to neuromorphic sensory network. Highly distributed synaptic sensory neurons are more readily driven by portable, distributed, and ubiquitous power sources. Here, we report a contact-electrification-activated artificial afferent at femtojoule energy. Upon the contact-electrification effect, the induced triboelectric signals activate the ion-gel-gated MoS_2_ postsynaptic transistor, endowing the artificial afferent with the adaptive capacity to carry out spatiotemporal recognition/sensation on external stimuli (e.g., displacements, pressures and touch patterns). The decay time of the synaptic device is in the range of sensory memory stage. The energy dissipation of the artificial afferents is significantly reduced to 11.9 fJ per spike. Furthermore, the artificial afferents are demonstrated to be capable of recognizing the spatiotemporal information of touch patterns. This work is of great significance for the construction of next-generation neuromorphic sensory network, self-powered biomimetic electronics and intelligent interactive equipment.

## Abstract (web summary)

Low power electronics endowed with artificial intelligence and biological afferent characters are beneficial to neuromorphic sensory network. Here, the authors report contact-electrification-activated artificial afferent at femtojoule energy, which is able to carry out spatiotemporal recognition on external stimuli.

## Introduction

With the rapid development of intelligent human-machine interfaces^1,2^, adaptive and highly distributed sensors, controllers, and actuators are commonly required to work synergistically to complete the functions of linkage, communication, and interaction with human beings^3^. More advanced and intelligent sensory networks call for neuromorphic sensory devices and bioinspired interactive systems similar to human brains, targeting the implementation of disordered, event-driven, parallel, and distributed computations^4–6^. It is believed that sensor networks endowed with biological afferent characters are promising for solving more complicated problems of reality, such as facial recognition, image understanding, fuzzy algorithms, and adaptive control^6^. Aiming at this goal, replicating the functionality of the human somatosensory system (composed of a network of distributed receptors, neurons, and synapses) is of great significance to endow electronic sensors, communicators, and actuators in sensory network with biological intelligence. The emerging field of synaptic electronics^7^ has been proposed as a surprising new way to conduct neuromorphic computing; synaptic electronics is a class of artificial devices that exhibit synaptic behavior similar to synapses in the nervous system^8^. Among various synaptic electronics^9^, three-terminal transistors with more analogous neuromorphic configurations and comparable artificial synaptic plasticity^10–20^ have been intensively investigated to simulate synaptic functions and construct sensory neurons^21,22^

For intelligent sensor networks, the highly distributed synaptic sensory neurons are more readily driven by portable, distributed, and ubiquitous power sources^23^. From the aspect of the distributed energy supply for a sensory network, a triboelectric nanogenerator (TENG) is versatile and thus able to effectively convert different types of mechanical energy into electricity from the ambient environment^24^. Based on the triboelectrification, which is a universal and ubiquitous effect with abundant material choices^25^, a TENG is capable of working with a micro/nano power supply, self-powered system, high voltage source, and blue energy^23^. In particular, the coupling effect between the triboelectric potential and semiconducting transport properties offers an active and direct linkage between the external mechanical motions and electrical output signals^26–28^, which can be potentially utilized to mimic the function of biological sensory neurons or afferent nerves. The external mechanical action imposed on TENG can be readily converted into voltage spikes (i.e., action potentials), captured by the synaptic device, and recognized with encoded input spatiotemporal features to deliver the feedback or regulation instructions. Furthermore, the converted mechanical energy can be used to drive the artificial sensory neuron in a self-powered fashion, which can significantly decrease the energy dissipations. Given the need for low-power-consuming artificial neural networks, pursuing energy-autonomous sensory neurons is highly desired to develop revolutionary neuromorphic systems.

Here, we present a contact-electrification (or triboelectrification)-activated artificial afferent neuron at femtojoule energy. The artificial afferent includes a self-activation component and a synaptic transistor to mimic the function of the human perception system. Originating from the charge transfer during contact-electrification (CE), the induced triboelectric signals activate the postsynaptic transistor and endow the artificial afferent with adaptive capacity to carry out spatiotemporal recognition on external stimuli, such as displacements, pressures, and touch patterns. The CE-activated artificial afferents are also capable of establishing dynamic logic and recognizing the frequency/magnitudes of external actions. The energy dissipation of the CE-activated artificial afferent has been significantly reduced to the femtojoule level (11.9 fJ per spike). The recognition of spatiotemporal touch patterns has also been successfully demonstrated to trigger corresponding LED logic as virtual excitations in the cerebral cortex.

## Results

### Design of the CE-activated artificial afferent

**Fig. 1:**
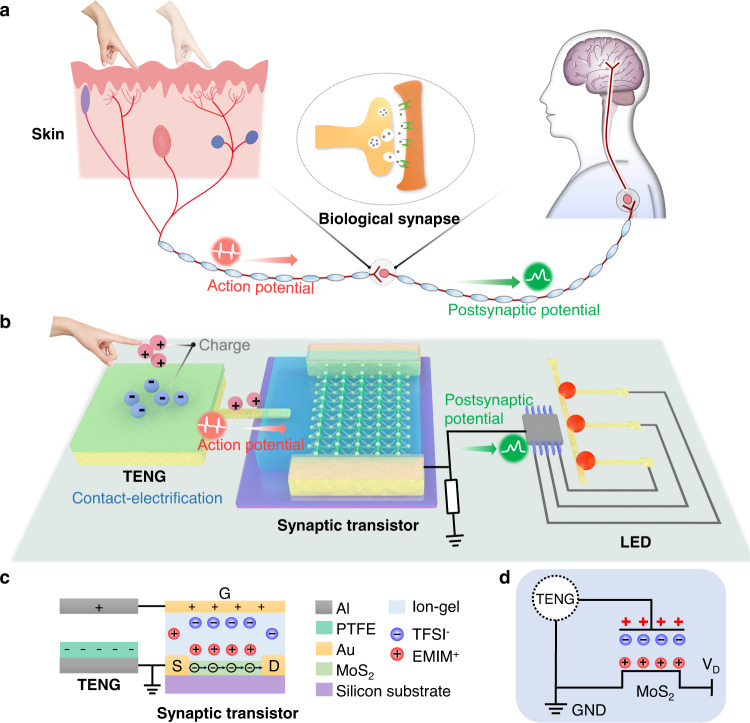
Biological afferent nerve system and CE-activated artificial afferents. **a** The basic procedure of the postsynaptic current activated by external stimulation in the biological afferent nerve system. The external stimulation initiates an action potential in the sensory neuron. The action potential propagates along with the nerve fiber and leads to a potential change in the adjacent nerve cell (i.e., activating postsynaptic current). **b** Schematic illustration of the CE-activated artificial afferent. It includes a self-activation component, a synaptic transistor, and a functional circuit. **c** Cross-sectional view of the CE-activated MoS_2_ synaptic transistor and illustration for each component. **d** Circuit diagram of the CE-activated artificial afferents.

Inspired by the afferent nerve system that the biological stimuli-receptors capture the external touch/stretch/stress/temperature/humidity stimulus and trigger the action/postsynaptic potential to be delivered via dorsal column-medial lemniscus pathway (Fig. 1a)^29–34^, we fabricate the contact-electrification-activated artificial afferents to mimic the function of biological afferent nerves. The triboelectric potential induced by contact electrification activates the synaptic transistor and delivers the relevant mechanical information to functional end terminals (Fig. 1b). Here, the triboelectric potential plays two critical roles: (i) it acts as the power source to drive (gate) the synaptic transistor (Fig. 1c, d); and (ii) it directly correlates the spatiotemporal information of the external stimuli with output signals (i.e., PSCs).

The self-activation component relies on the TENG technique. It is arbitrary with multiple geometries and abundant materials (any material capable of contact-electrification) to reflect spatiotemporal information such as displacement, pressure, or touch pattern. The self-activation component in optional contact-separation (CS), sliding or single-electrode mode enables the recognition of displacement or touch patterns (Supplementary Fig. 1a–c). It is also applicable to the design of a three-point bending architecture (Supplementary Fig. 1d) to monitor external pressure^35–38^, by correlating the pressure information with the displacement in a nonlinear relationship. The external stimuli induce triboelectric potentials through the TENG technique to activate the synaptic transistor, which is a self-powered process according to contact-electrification and electrostatic induction. The driving triboelectric potentials accompanied by CE can also be quantified by the output signals from synaptic transistors, i.e., the external mechanical stimuli are quantifiable by the artificial afferent neuron.

The synaptic transistor is an ion-gel-gated MoS_2_ field-effect transistor (FET, Supplementary Fig. 2). The wide band gap and atomically thin body of MoS_2_ (Supplementary Fig. 3) can efficiently reduce the direct source-drain tunneling current and improve the transport properties in the channel for high-performance neuromorphic signal transmission and low energy dissipation^28^. The ion gel is intrinsically a solid-state electrolyte (Supplementary Fig. 4), in which the migration and distribution of ions determine the spatiotemporal postsynaptic electric signals (Supplementary Fig. 5)^5,39,40^. Based on the ion-gel-gated FET, we can achieve the gradual decay behavior of the postsynaptic current (75 ms, Supplementary Fig. 5b) according to the slow migration of ions during the unconventional form of the electrical double layers (EDLs)^4,40^. This process is critical for the ion-gel-gated synaptic transistors to imitate the working process of biological synapses (Supplementary Fig. 6).

In CE-activated artificial afferents, we consider the contact/separation or sliding forward/backward cycle actions as the activation input pulses. The ion gel (ionic conducting but electrical insulating) can act as a buffer layer to rectify/modify the intrinsically opposite paired pulses of the TENG to minimize (or remove) the influence of the second opposite pulse. Thus, the induced triboelectric voltages can be efficiently coupled to the FET channel through the ion gel and behave like an equivalent gate voltage supply (i.e., triboelectric gate potential, Supplementary Fig. 7)^26,28^. Through CE-activation, the artificial afferents (synaptic transistors) show representative biological synaptic behavior, such as the decay time, excitatory postsynaptic current (EPSC), and paired-pulse facilitation (PPF). They are also capable of recognizing spatiotemporal information such as displacements and touch patterns. As a proof of concept, we design a voltage amplifier and processing circuit to convert the corresponding PSCs (related to the spatiotemporal touch information) into output trigger voltages to drive LEDs in relevant logics (representing the virtual excitations in the cerebral cortex). This work demonstrates a coupling of triboelectric charges, ions, and electrons triggered by touch/sliding/pressure, each of which can be converted into triboelectric signals and induce corresponding PSCs in a self-powered fashion to reflect the spatiotemporal information of external mechanical stimuli, i.e., contact-electrification-activated afferent neurons. In contrast to the previous literature, by applying a stimulating or driving voltage to imitate synaptic behaviors^9,41,42^, the triboelectric potential triggered by external displacement, pressure, or touch can be considered as a self-powered action potential to complete the artificial somatosensory system. It offers a more direct coupling between external stimuli and PSCs, meanwhile greatly decreasing energy consumption.

### Typical synaptic characteristics of the CE-activated artificial afferent

**Fig. 2:**
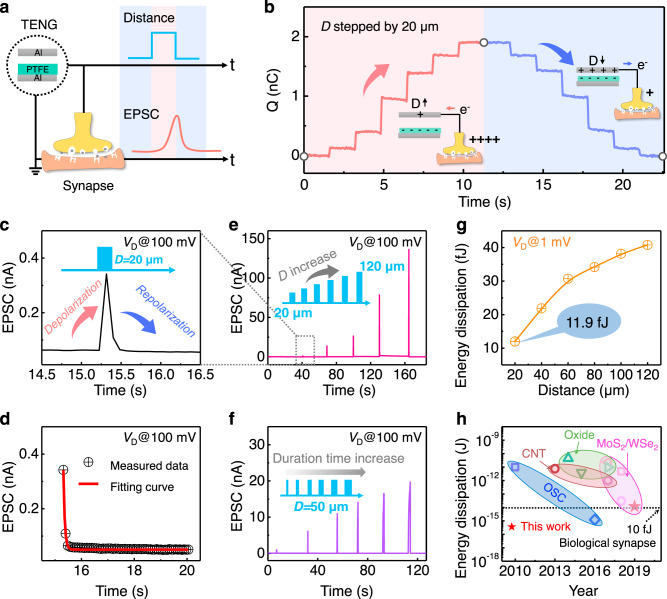
Typical synaptic characteristics of the CE-activated artificial afferent. **a** Schematic illustration of an EPSC activated by a single CS action. **b** The transferred charges of the CE-activated MoS_2_ synaptic transistor within a cycle of contact-separation. **c** The EPSC responses under one CS action (*D* = 20 μm). **d** The decay phenomenon fitting of the EPSC with the exponential decay model (Supplementary Note 3). **e** The EPSCs under different CS action distances. Inset: *D* increases from 20 to 120 μm. **f** The EPSC responses under different CS action durations (*D* = 50 μm). Inset: Illustration of the increase in duration. **g** The energy dissipation vs. distance. **h** Comparison of the energy dissipation per spike for different types of artificial synapses. The dissipation is 11.9 fJ in the CE-activated artificial afferent (red star).

We take the CE-activated artificial afferents in contact-separation mode (CS mode) as an example to demonstrate the neuromorphic behaviors. The CE-activation part is composed of a polytetrafluoroethylene (PTFE) film and an Al electrode (selected according to whether it has enough different electronegativities in the triboelectric series). During the CS process, the induction charges on the Al/PTFE contribute to a Maxwell displacement current and result in a variable electric field (i.e., triboelectric potential or action potentials) related to the separation distance (*D*, considered as the presynaptic signal in spatial fashion). With PTFE/Al electrode in the CE-activation part connected to the common ground with the source electrode, the triboelectric potential can be effectively coupled to the synaptic transistor and trigger the EPSC (Fig. 2a, Supplementary Fig. 8). During the CS process, the transferred charges (*Q*) are critical in affecting the EPSC behavior and are closely related to the separation distances (Fig. 2b). A separation distance of 20 μm leads to positive charges of ~0.3 nC transferring to the gate of the synaptic transistor, equivalent to applying a gate voltage of 0.125 V to drive the transistor (Supplementary Fig. 9).

The typical EPSC behavior of the CE-activated artificial afferent is characterized in Fig. 2c. A capacitor with 1 nF is connected in parallel as a buffer to ensure steady triboelectric potential coupling (detailed discussions in Supplementary Note 1, Supplementary Fig. 10, and Supplementary Fig. 11). The separation displacement, acting as the presynaptic input, electrifies the friction layers and couples the triboelectric potential to the ion-gel-gated MoS_2_ synaptic transistor. It then induces anion/cation migration to form EDLs in the ion gel, efficiently shifts the Fermi level of the MoS_2_ channel, and triggers the EPSC (Supplementary Note 2 and Supplementary Fig. 12). An input displacement at 20 μm results in an EPSC of 0.34 nA, which gradually decays back to the initial value during the contact process between two friction layers of the TENG (Fig. 2c). The separation/contact cycle action (or sliding forward/backward in sliding mode) functionalizes as the activation input pulse of the artificial afferent. The decay time ($\tau$) for the CE-activated EPSC (0.34 nA) is estimated to be ~54 ms, indicating that the feature time of ion migration is ~54 ms (Fig. 2d). Notably, the decay time under CE-activation is similar to the intrinsic EPSC (0.87 nA) of the ion-gel-gated synaptic transistor (75 ms, Supplementary Fig. 5b). Compared with previous reports, the decay time of our device triggered by a single pulse is in the range of the sensory memory stage (Supplementary Note 4 and Supplementary Fig. 13a). A survey of the decay time of the previous literature has been listed in Supplementary Table 1 and Supplementary Fig. 13b. The achieved decay time of 75 ms may fluctuate according to the amplitude, duration time, and pulse number of the external stimulation. It has the potential to transform from sensory memory to a short-term memory model. Further engineering on the architecture of the synaptic transistor or developing proper training methods is probably to realize the long-term memory model. The relatively longer decay time compared with that of the synapses in biological afferent nerves (1.5 to 5 ms) presumes the imitation of the learning or memory behavior in the future.

The CE-activation behavior is comparable with the EPSC activation process in a biological synapse. First, a larger displacement (reflecting the spatial information) means a higher action potential in the neural system, which attracts more ions in the synaptic transistor to contribute to the EDLs and induce higher EPSC. As the separation distance increases from 20 to 120 μm, the peak value of the EPSC increases from 0.34 to 136 nA (Fig. 2e). The action potential of the displacement spike (20 μm) gives rise to a similar EPSC at a gate voltage spike of ~0.5 V (Supplementary Fig. 5c).

Second, the duration time of the displacement reflects the temporal information of the external stimuli. To check the ability to recognize the temporal information based on the mechanical displacement, we hold the CE component in the separated state (*D* = 50 µm) for different amounts of time and monitor the corresponding EPSCs of the artificial afferent. The peak current of the EPSC increases from 0.97 to 20 nA as the duration time of the separated state increases from 0.08 to 1.75 s (Fig. 2f). The longer duration of the separation state gives enough time for the cation accumulation at the interface, more effectively enhances the electron transport in the MoS_2_ channel, and leads to a higher EPSC. Notably, the peak value of the EPSC shows a linear increment (for a CS spike duration within 0.6 s) with a subsequent saturation tendency (for a duration ranging from 0.6 to 1.75 s) (Supplementary Fig. 14). The gradually saturated tendency is attributed to the ion having enough time to form EDLs over longer durations, which is consistent with biological synaptic behavior with gradually saturated neurotransmitters under continuous biological action potentials.

Third, the low power consumption of ~10 fJ per spike is the key to realizing complex computations assisted with millions of synapses^14,43^. To reduce the power consumption of the artificial afferents (or synapses), it is generally critical to reducing the operating voltage and leakage current of the synaptic transistor. The CE-activated artificial afferent is particularly promising for ultralow power consumption: (i) the gate supply is completely replaced by the triboelectric potential; (ii) the atomic thickness of the MoS_2_ channel permits a low operating voltage; and (iii) a thin encapsulation layer on the source-drain electrodes ensures the ultralow gate leakage current^28^. With the drain voltage scaling down to even 1 mV, the MoS_2_ channel still maintains excellent transfer properties and typical synaptic pulse output behaviors with efficient gate modulation ability (Supplementary Fig. 15). The power consumption of this CE-activated artificial afferent can be significantly reduced to 11.9 fJ per spike (Fig. 2g, evaluated from Supplementary Fig. 15c, and Supplementary Note 5), which is comparable to the human synapse (10 fJ), highly approaches the organic nanowire transistor (1 fJ)^14^, and precedes most of the artificial counterparts (e.g., the organic and oxide semiconductor^11,22,44,45^, CNT^46,47^, and 2D materials^4,5,19^ based synaptic transistor) in previous literatures (Fig. 2h, Supplementary Table 1). The results show that our device has great potential to simulate the low-power-consuming neuromorphic bioelectronic devices with multiple functions. Note that our estimates for the dissipation energy are based on our specific model for the dissipation energy described above. It is possible that within practical applications additional dissipating channels are present, which can increase the dissipation energy. For instance, the back-end processing circuit and flash LED circuits are part of demonstrations instead of the necessary component of the CE-activated artificial afferent. Their energy dissipations are excluded for evaluation of the ultralow-power consumption of CE-activated artificial afferent.

According to the bijective relation between EPSCs and separation distances (or duration time), the EPSC signals are the direct reflection of the external displacements, which is applicable when deducing the applied mechanical displacements. The sensitivity can be divided into two regions: 7 μm^−1^ and 45 μm^−1^ for region *I* and region *II*, respectively (Supplementary Fig. 16). The functional integration of distance recognition and signal differentiation is the fundamental feature of the CE-activated artificial afferent. The artificial afferent in different working modes (sliding and single-electrode mode) offers optional ways to recognize different types of displacements or touch patterns as needed (Supplementary Fig. 17, 18). The corresponding sensitivity in the sliding and single-electrode mode is 0.52 μm^−1^ and 17.8 μm^−1^, respectively. We can also change the geometric design of the self-activation component with a three-point bending structure for pressure recognition (sensitivity is 12.3 kPa^−1^, Supplementary Fig. 19 and Supplementary Note 6).

### Advanced synaptic characteristics of the artificial afferent

**Fig. 3:**
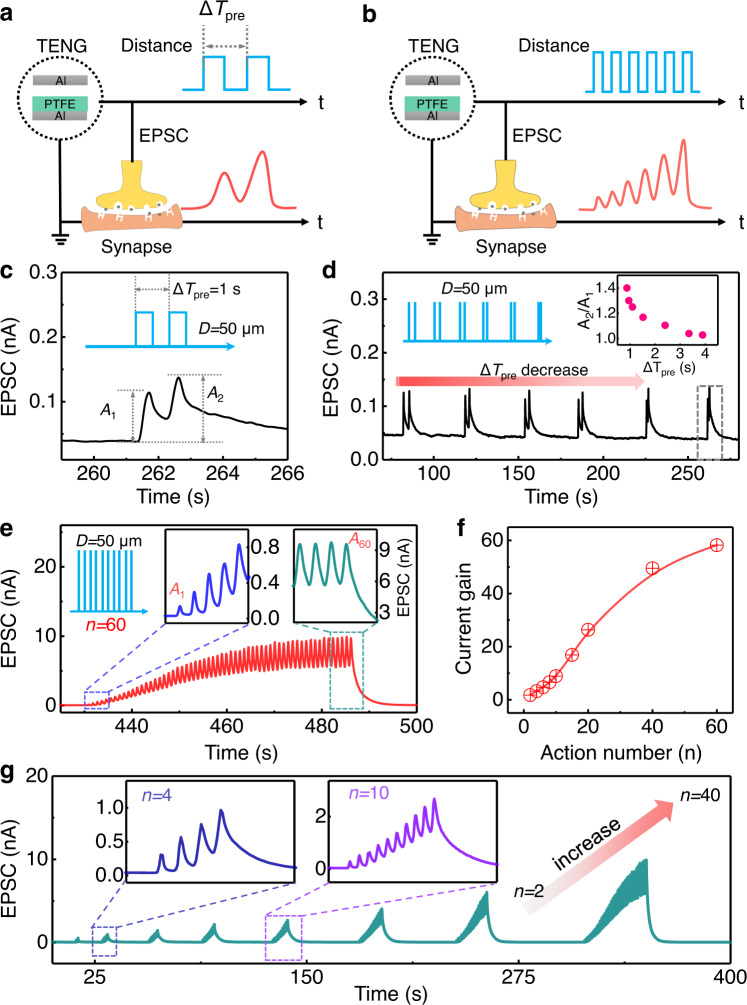
The basic synaptic plasticity of CE-activated artificial afferents. Schematic illustration of EPSCs activated by (**a**) paired CS actions and (**b**) multiple CS actions. **c** The typical EPSC responses under paired CS actions (interspike interval, △*T*_pre_ = 1 s). *A*_1_ and *A*_2_ represent the amplitudes of the first and second EPSCs, respectively. **d** A series of EPSC responses activated by paired CS actions with different interspike intervals, with *D* = 50 μm. The interspike interval decreases from 4 s to 1 s. Inset: the PPF index (defined as the ratio of *A*_2_*/A*_1_) vs. the interspike interval. **e**, The EPSC responses under 60 CS actions. Inset: an illustration of 60 CS actions (left) and the first (*A*_1_, middle) and last (*A*_60_, right) current peak of the EPSCs. **f** The current gain (defined as the ratio of *A*_n_ */A*_1_) vs. action number. **g** Real-time EPSC responses to multiple CS actions; the number of actions (*n*) increases from 2 to 40. Inset: the EPSC responses under *n* = 4 (left) and *n* = 10 (right).

To further characterize the temporal recognition capacity of CE-activated artificial afferents, we apply paired and multiple CS actions as the input stimuli pulses and investigate the corresponding EPSCs (Fig. 3a, b). Under the stimuli of consecutive and paired CS mechanical actions, the self-activation artificial afferent represents typical paired-pulse facilitation (PPF) behavior, i.e., the EPSC evoked by the second spike is increased when it closely follows the previous spike (Fig. 3c). In a typical PPF, the peak current of the EPSC activated by the second contact-separation spike (*A*_2_ = 0.14 nA) is larger than that of the first one (*A*_1_ = 0.1 nA). The demonstrated PPF behavior is a form of short-term potentiation (STP) synaptic plasticity, which is important for decoding temporal information and neuromorphic computations in an artificial biological system. The PPF index (defined as *A*_2_/*A*_1_), which evaluates the facilitation degree of the synapse, is closely related to the interval time between the presynaptic stimuli pulses (△*T*_pre_, defined by the interspike interval time between the paired CS actions). A series of EPSC responses activated by paired CS spikes (*D* = 50 μm) with different △*T*_pre_ values is shown in Fig. 3d. The CE-activated artificial afferent shows an increasing trend of the PPF index with shorter △*T*_pre_ (inset of Fig. 3d), which is attributed to the fact that the ions contributing to the formation of EDLs do not have enough time to drift back and recontribute to the formation of the EDLs with the ions triggered by the second CS spike. The tunable capacity of the PPF index initialized by the paired CS actions demonstrates that the CE-activated artificial afferent has excellent short-term synaptic plasticity, which is beneficial for neural information computation and processing.

The CE-activated artificial afferents also possess the function of further potentiation plasticity under multiple consecutive CS action pulses (*D* = 50 μm, Fig. 3e), represented by a series of triggered EPSCs. The exertion of 60 CS-spike cycles increases the peak current of the EPSCs from 0.03 to 9.7 nA. The multiple consecutive CS spikes trigger multiple triboelectric pulses and continuously activate the MoS_2_ channel through the ion gel. According to the gradual saturation of ions when constructing EDLs, the EPSC varies from an obvious increment tendency (initial excitation stage) to a subdued state (the last several cycles, inset of Fig. 3e). This behavior is similar to that of the saturated neurotransmitters under multiple presynaptic pluses. The current gain, defined as *A*_n_/*A*_1_ (*A*_1_ and *A*_n_ represent the peak current of the first and last EPSC, respectively), is closely related to the CS action numbers (Fig. 3f). This relation exists because the number of CS actions directly determines the number of ions when constructing EDLs to modulate the MoS_2_ Fermi level. The CE-activated artificial afferent shows gradually enhanced potentiation plasticity in real-time with increased stimuli action numbers (from 2 to 40 cycles, Fig. 3g). Excellent reliability and durability of the CE-activated artificial afferents with no obvious baseline drift have also been demonstrated in Supplementary Fig. 20 and Supplementary Fig. 21, which promises more availability and feasibility in practical applications.

**Fig. 4:**
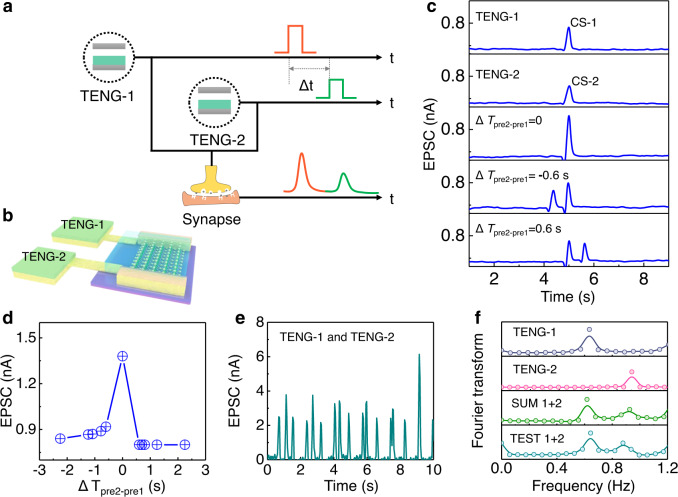
The advanced spatiotemporal synaptic characteristics of CE-activated artificial afferents. **a** Schematic illustration of EPSCs activated by dual-TENGs. **b** Schematic illustration of CE-activated artificial afferents in dual-TENG mode. **c** EPSCs activated by single and paired CS actions with spatiotemporal information. **d** The recorded second EPSCs vs. Δ*T*_pre2-pre1_ (interval time between the first and second CS actions). **e** The recorded EPSC responses to TENG-1 and TENG-2 simultaneously. **f** The extracted frequencies of the TENG-1 and TENG-2 activations after Fourier transform. The frequencies of the two activation components are clearly extracted from Fig. 4e and Supplementary Fig. 23.

Spatiotemporally correlated stimulation from multiple presynaptic terminals can be used to activate postsynaptic currents to establish dynamic logic in an artificial neural network^14,22^. The CE-activated artificial afferent is also capable of simulating the spatiotemporal dynamic logic by utilizing the planar dual-gate mode synaptic transistor (with two TENGs coupled to a single MoS_2_, Fig. 4a, b), which is similar in geometry with the fore-end of the biological myelinated nerve fiber (i.e., heminode)^48–50^.

Two distinguishable CS actions with an interval time (△*T*), considered as the key spatiotemporal trigger sources, are applied to the artificial afferent. The different contact areas of the two TENGs (TENG-1 and TENG-2) induce different output voltages (Supplementary Fig. 22). The CS spike of TENG-1 (*D* = 30 µm) triggers EPSC-1 with a peak current of ~0.74 nA as the first presynaptic pulse, while the CS spike of TENG-2 (*D* = 20 µm) triggers EPSC-2 with a peak current of ~0.6 nA as the second presynaptic pulse (Fig. 4c). When the two CS spikes are applied sequentially with an interspike interval (△*T*_pre2-pre1_), the influence of the second presynaptic pulse on the first one is evaluated with the recorded first EPSC. When △*T*_pre2-pre1_ = 0, EPSC-1 and EPSC-2 are triggered simultaneously, resulting in a maximum EPSC of ~1.06 nA in the postsynaptic terminal. When △*T*_pre2-pre1_ = −0.6 s, the CS-2 will superimpose with CS-1 and contribute to EPSC-1, leading to an enhanced EPSC-1. When △*T*_pre2-pre1_ = 0.6 s, CS-2 is applied later than CS-1 and delivers no contribution to EPSC-1, leading to an unchanged EPSC-1. The measured EPSC amplitude at the end of the TENG-1 CS spike (set as *t* = 0, namely, when the summed EPSC is recorded) is plotted vs. △*T*_pre2-pre1_ to understand the influence of the CS spike on a spatiotemporally correlated presynaptic terminal (Fig. 4d). The amplitude of the EPSC at *t* = 0 is consistent with the amplitude of EPSC-1 if EPSC-2 is triggered afterward (△*T*_pre2-pre1_ > 0). In contrast, when EPSC-2 is triggered before EPSC-1 (△*T*_pre2-pre1_ < 0), the EPSC at *t* = 0 is influenced by the superimposition of EPSC-1 and the remaining EPSC-2 activated by CS-1 and CS-2, respectively. The EPSC dynamic logic of the self-activated synaptic transistor in dual-gate mode represents a nonlinear spatiotemporal relationship (Fig. 4d). This is similar to the response of biological hippocampal CA1 pyramidal neurons under spatiotemporally correlated stimuli from different presynaptic terminals.

Except for the dynamic logic correlated with the interval time, it is also of great significance to recognize the frequency and magnitudes of external actions with the CE-activated artificial afferent. We monitor the real-time EPSCs simultaneously activated by the two TENGs at different contact-separation frequencies (Fig. 4e). The combination of the EPSC coordinates of TENG-1 (*D* = 0.3 mm) and TENG-2 (*D* = 0.2 mm) is shown in Supplementary Fig. 23. Both the frequencies and magnitudes of the displacements applied to the CE-activated artificial afferent can be recognized after Fourier transforms according to the different spatiotemporal patterns of the postsynaptic currents (Fig. 4f). The monitored results after the Fourier transform are consistent with both the initial and combined data, representing the excellent temporal recognition ability of the artificial afferent. The demonstrated MoS_2_ synaptic transistor can combine with and recognize the triboelectric potential inputs from two pre-TENGs, exhibited as the EPSCs comprising the temporal information at two corresponding frequencies. The CE-activated artificial afferent in dual-gate (or multigate) mode mimics the way multiple presynaptic neurons input action potentials to the dendrites of a postsynaptic neuron, which is critical for abundant tactile sensation by the biological SA-I afferent nerve in advanced animals^48^. Furthermore, the contact-electrification induces coupling of the triboelectric potential to the MoS_2_ postsynaptic transistor in a self-activated way without any gate supply, which is beneficial for the low-power-consuming decoding of mechanosensation signals containing spatiotemporal information.

### Dynamic logic recognition of spatiotemporal touch patterns

**Fig. 5:**
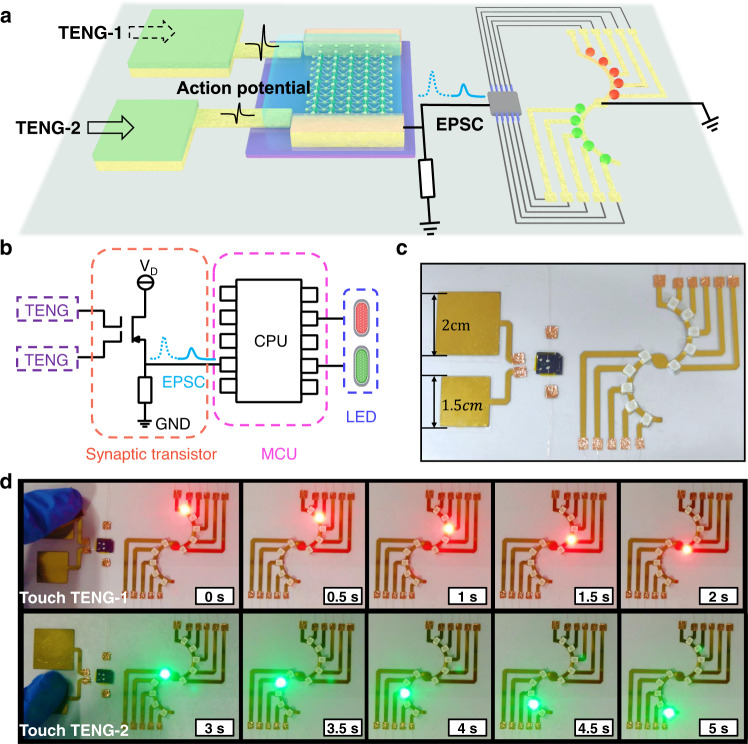
Demonstration of the dynamic logic of the CE-activated artificial afferent. **a** Schematic illustration diagram, (**b**) a simplified circuit diagram, and (**c**) a photo of the artificial afferent for dynamic logic demonstration. **d** Red LEDs triggered by CS action from TENG-1 (top) and green LEDs triggered by CS action from TENG-2 (bottom).

As a proof of concept, the dynamic logic function of the artificial afferent is demonstrated on a flexible substrate with the assistance of flash LED circuits. The system consists of a CE-activated artificial afferent neuron (Supplementary Fig. 24), a microcontroller unit (MCU), and two groups of LEDs in series (Fig. 5a). The dynamic logic is reflected through the flash sequences of the LEDs by recognizing different afferent signals induced by contact-electrification. When two different triboelectric potentials are coupled to the synaptic transistor (rectifier and filter), different EPSCs are triggered and converted into relevant bias voltage outputs through a bleeder circuit (Fig. 5b and Supplementary Fig. 25). The voltage outputs captured by the processing circuit are recognized as the threshold signals and drive the corresponding flash LED circuit (Supplementary Fig. 26). When a touch action is applied to TENG-1 (2 × 2 cm^2^), the triggered voltage is >2 V with a corresponding EPSC > 30 nA. The converted voltage trigger signal (>0.5 V) through the bleeder circuit can be acquired and recognized to induce a series of red lights to illuminate (Fig. 5d). When a similar touch pattern is applied to TENG-2 (1.5 × 1.5 cm^2^), the triggered voltage is <2 V with corresponding EPSC < 30 nA and trigger voltage <0.5 V, which can induce a series of green lights to illuminate (Fig. 5e, Supplementary Movie 1). More complex external touch patterns applied in the sequence are also distinguishable by the CE-activated artificial afferent (Supplementary Movie 2), representing the excellent capacity of the self-activated artificial afferent to recognize the spatiotemporal information of touch patterns.

## Discussion

In summary, we successfully demonstrate contact-electrification-activated artificial afferents. According to the CE effect, the induced triboelectric potential can effectively activate the postsynaptic transistor and endow the artificial afferent with the good capacity to carry out spatiotemporal recognition on external stimuli, including displacements, pressures, and touch patterns. The CE-activated artificial afferents are also capable of establishing dynamic logic and recognizing the frequency/magnitudes of external actions. The energy dissipation of the contact-electrification-activated artificial afferent is significantly reduced to 11.9 fJ per spike owing to the removal of the gate voltage supply, utilization of an EDL gating, and excellent electrical performance of MoS_2_. The recognition of spatiotemporal touch patterns is also successfully demonstrated on a flexible substrate. This work represents a promising strategy for the development of next-generation biomimetic electronics, low-power-consuming neuromorphic devices, direct-interactive electronic prostheses, and even neurorobotics.

The proposed CE-activated artificial afferents represent a direct interaction between the mechanical actions and postsynaptic behaviors in an active and self-powered way. It is intrinsically a highly efficient coupling between the triboelectric potential and semiconducting transport properties through ultrahigh capacitive EDLs, namely, tribo-iontronics. It offers a universal way to construct a broad category of contact-electrification-activated neuromorphic electronic devices with low energy dissipation, e.g., multimodal optoelectronic synapses, mechanically programmed artificial memory neurons, multifunctional sensory synapses, and biomimetic motor neurons. Based on the CE-activated artificial afferents, the diversified structure design of the self-activation component is applicable and customizable to different circumstances on demand. The synaptic device is also ready to be extended to other types of transistors, e.g., EDL transistors, proton conductor derived transistors, floating-gate transistors, and suspended-gate transistors. Furthermore, contact electrification is ubiquitous; the CE-activation delivers an efficient way for readily harvesting random and distributed mechanical energy from surroundings to solve the disordered and fuzzy problem in the neuromorphic sensory network, implying the theory of entropy (describing thermodynamic disorders) relates to the disordered/distributed/self-powered sensing from the aspect of energy utilization. The CE-activated neuromorphic afferents at femtojoule energy with broad application prospects pave the way for neuromorphic computation with high efficacy, multiple modalities of mechanosensation, and ultralow-power consumption.

To further develop the CE-activated afferent in lower dimension (e.g., micrometer scale), active-matrix design of flexible synaptic transistor array is an optimal option; for the nanometer scale applications, atomic force microscopy (AFM) and Kelvin probe force microscopy (KPFM) are efficient means to investigating the electron transfer, surface potential, and triboelectric charge decay in nanometer-scale contact electrification, which offers a potential way to developing a nanoscale neuromorphic device with in-memory computing functions (Supplementary Note 7). Another possible concern on the CE-activated afferent is the susceptibility of utilized ion gel to surrounding moisture/temperature variations, which may not be an absolute drawback yet can be appropriately combined with the CE-activation sensing and extended to multimodal humidity/thermal/mechano-sensation. Elaborate selection or synthesis of hydrophobic and chemically stable ionic liquid paired with proper gelation polymers or encapsulating elastomers with excellent extreme-temperature tolerance can also effectively alleviate the humidity/temperature susceptible issues (Supplementary Note 8). The demonstrated CE-activated neuromorphic afferents are qualified to be the trendsetter in the development of intelligent Internet of Things and artificial nerves to overcome the bottleneck of von-Neumann architectures.

## Methods

### Materials preparation

Triangle-shaped single-crystal MoS_2_ was grown on a Si wafer assisted with a three-temperature-zone chemical vapor deposition (CVD) system. The precursors (Sulfur, Alfa Aesar 99.9%, and molybdenum trioxide (MoO_3_), Alfa Aesar 99.999%) and SiO_2_/Si substrate were loaded in the growth Zone I, II, and III, respectively. The working temperatures of Zone I, II, and III were 150 °C, 560 °C, and 850 °C, respectively. During the growth process, argon was used as the carrier vapor of the precursors maintained at a flow rate of 50 sccm to provide an inert atmosphere. The pressure in the quartz tube was kept at 1.0 torr during the synthesis process. After the synthesis process of MoS_2_, the tube was quickly cooled down to room temperature.

### Device fabrication

To fabricate the MoS_2_ synaptic transistor, the MoS_2_ grown on SiO_2_/Si substrate was firstly spin-coated with poly (methyl methacrylate) (PMMA). Next, the patterns of co-planar source/drain and gate electrode were defined by standard e-beam lithography (EBL). Cr/Au (10/40 nm) electrodes were then deposited at a rate of 1 Å·s^−1^ through thermal evaporation followed by a lift-off process to form source-drain contacts and gate electrode for the MoS_2_ transistor. A thin layer of PMMA (150 nm) was patterned on source-drain electrodes by a second EBL process to decrease the leakage current. After that, a UV-curable ion gel as the dielectric layer was patterned on the MoS_2_ channel and a portion of the gate electrode to achieve the synaptic transistor. The ion gel was composed of the polymer framework of poly (ethylene glycol) diacrylate (PEGDA), the photo cross-linker of 2-hydroxy-2-methylpropiophenone (HOMPP), and the ion liquid of 1-ethyl-3-methylimidazolium bis(trifluoromethylsulfonyl) imide ([EMIM][TFSI]), at a weight ratio of 8:2:90. The triggering of the flash LED circuits by spatiotemporal touch patterns was demonstrated on a flexible polyethylene terephthalate (PET) substrate. The Au electrodes for the TENG touch pads (friction layer) and necessary electrical circuits were patterned by standard photolithography and wet etching process. Other components were mounted on the PET substrate.

### Characterization

The Raman spectrum was measured by a HORIBA/LabRAM HR Evolution spectrograph. The wavelength of the excitation laser was 532 nm. All the electrical characterizations for the MoS_2_ synaptic transistor were carried out using a semiconductor parameter analyzer (Agilent B1500A) in a probe station. The displacement of TENGs was controlled by a linear motor. The output performance of TENGs was characterized by Keithley 6514 electrometer.

### Reporting summary

Further information on research design is available in the Nature Research Reporting Summary linked to this article.

## Supplementary information

Supplementary Information

Description of Additional Supplementary Files

Supplementary Movie 1

Supplementary Movie 2

Reporting Summary

## Data Availability

All data supporting this study and its findings are available within the article and its Supplementary Information or from the corresponding author upon reasonable request.
