# Supplementary material for: Contact-electrification-activated artificial afferents at femtojoule energy
Source: Nat Commun. 2021 Mar 11;12:1581. doi: 10.1038/s41467-021-21890-1 (PMC7952391; doi:10.1038/s41467-021-21890-1)
Supplement: Supplementary file 1 — Supplementary Information [file 41467_2021_21890_MOESM1_ESM.pdf]

Supplementary Materials for

Contact-Electrification-Activated Artificial Afferents at Femtojoule Energy

Jinran Yu,[1,2,Δ] Guoyun Gao,[1,2,Δ] Jinrong Huang,[1,2,Δ] Xixi Yang,[1,2] Jing Han,[1,2] Huai Zhang,[1,2] Youhui Chen,[1,2] Chunlin Zhao,[1,2] Qijun Sun,[1,2,3]* Zhong Lin Wang[1,4]*

[1]*Beijing Institute of Nanoenergy and Nanosystems, Chinese Academy of Sciences, Beijing 100083, China*
[2]*School of Nanoscience and Technology, University of Chinese Academy of Sciences, Beijing 100049, P. R. China*
[3]*Center on Nanoenergy Research, School of Physical Science and Technology, Guangxi University, Nanning, 530004, China*
[4]*School of Materials Science and Engineering, Georgia Institute of Technology, Atlanta, Georgia 30332-0245, United States*

[Δ]*These authors contributed equally: Jinran Yu, Guoyun Gao, and Jinrong Huang*

* To whom the correspondence should be addressed.
Email: sunqijun@binn.cas.cn; zhong.wang@mse.gatech.edu.

Keywords: artificial afferents, self-activation, synaptic transistor, contact-electrification, neuromorphic devices

Supplementary Note

Supplementary Note 1

Investigations on connected capacitors: The contact-electrification-activated MoS_2 synaptic transistor is an ion-gel-gated transistor capacitively coupled to a TENG. The TENG is generally regarded as a variable capacitor, while the ion-gel-gated transistor can also be regarded as a metal-insulator-semiconductor (MIS) capacitor. The capacitive coupling is essentially two capacitors connected in parallel. When there is no third capacitor connected in parallel, the charges induced by the triboelectric potential are all coupled to the MIS capacitor (*i.e.*, MoS_2 transistor), lead to the formation of EDLs and modulate the channel conductance. When there is an extra capacitor connected in parallel, as shown in Figure S11a, the tribopotential-induced charges are distributed between the third capacitor and MIS capacitor. The capacitor connected in parallel with a larger capacitance is allocated more triboelectric charges. Consequently, the number of triboelectric charges allocated to the MoS_2 transistor is less, resulting in a decreased I_D. According to Figure S11b to S11e, the current level gradually decreases as the connected capacitance changes from 0 to 4.7 nF. At a TENG displacement of 400 μm, the output I_D of the contact-electrification-activated MoS_2 synaptic transistor connected to a capacitor of 0, 0.47, 1, and 4.7 nF is 0.5 μA, 0.1 μA, 0.03 μA and 0.08 nA, respectively. Based on the above results, the output I_D can be effectively tuned by connecting different capacitors. More importantly, the EPSC (closely related to the output I_D) and the corresponding power dissipation of the synaptic device can be further optimized by choosing a suitable extra capacitor. In addition, the tribo-iontronic devices connected to an extra capacitor are found to be more robust during our testing. Through buffering by the extra capacitor, the breakdown of the transistor and other negative effects induced by the relatively high triboelectric pulses are greatly evitable. To validate the selection of 1 nF capacitor, corresponding charging curves of the TENG with different capacitors (0, 0.47, 1, and 4.7 nF) in parallel are measured in Figure S10. As shown in Figure S10 and Figure S11f, without the

external capacitor, the output voltage is too large for the synaptic transistor, which easily causes the electric breakdown of the transistor part. On the other hand, with a large capacitor, the output voltage will be tiny, which can not modulate the synaptic transistor effectively. Therefore, we reasonably select the connected capacitor of 1 nF for most of the characterizations in this work.

Supplementary Note 2

Working mechanism and the corresponding energy band diagram: We take the contact-separation mode as an example (Figure S12). In the initial state, the top Al layer is in full contact with the PTFE film ($D=D_0$). Owing to the different triboelectric polarities of the two friction layers, the electrons transfer from the top Al layer to the surface of the PTFE film, leaving positive net charges on the Al layer and negative net charges on the PTFE film. In this situation, the triboelectric potential input at the presynaptic terminal (Au gate) is 0 because the positive and negative charges on the two friction layers are balanced. The cations ($EMIM^+$) and anions ($TFSI^-$) in the ion gel are randomly distributed, and no current flows in the postsynaptic terminal (MoS_2 channel). When the top Al layer separates from the PTFE film (D increases from D_0 to D_1), the positively charged Al electrode attracts electrons from the presynaptic terminal of the MoS_2 synaptic transistor. Consequently, a positive triboelectric potential is generated at the presynaptic terminal, inducing the anions to migrate towards the ion gel/Au interface and the cations to migrate towards the ion gel/MoS_2 interface in the ion gel. The ions further accumulate upon the triboelectric potential and contribute to the formation of EDLs at the ion gel/Au and ion gel/MoS_2 interfaces. The accumulation of cations at the ion gel/MoS_2 interface induces the EPSC response in the MoS_2 channel. When the top Al layer returns to the original position (contact state, D, decreases from D_1 to D_0), the induced triboelectric charges are rebalanced, and the electrons flow back to the Au gate electrode. The presynaptic potential returns to 0, and the cations and anions gradually diffuse

back to the initial random distribution state. The measured output current exhibits a gradual recovery to the initial level, corresponding to the decay of the EPSC.

Relevant energy band diagram: At the initial contact state ($D=D_0$), no triboelectric potential is applied to the MoS_2 transistor. The cations and anions in the ion gel dielectrics are distributed in a random state, and no electrons are accumulated in the MoS_2 channel. The conduction band of MoS_2 is located in a high position, and the energy barrier height between the MoS_2 channel and the source electrode is relatively high. The limited electrons in MoS_2 under zero triboelectric potential gating are difficult to cross the energy barrier height and transport to the drain electrode. In this case, the MoS_2 transistor is in the off-state with low drain current. At the separation state ($D=D_1$), the positive triboelectric potential induces the cations to migrate towards the ion gel/MoS_2 interface and accumulates the electrons in MoS_2 channel. This leads to the downward bending of the energy band and decreases the energy barrier height between the MoS_2 channel and the source electrode. Thus, the accumulated electrons in MoS_2 channel are much easier to flow to the drain electrode. The MoS_2 is turned on and the drain current will be increased through the triboelectric potential gating.

Supplementary Note 3

Fitting the EPSC decay phenomenon: The decay time (τ) for this ion gel-induced EPSC decay phenomenon can be well fitted by using the exponential decay model, expressed as the following equation:

$$I(t) = I_{\infty} + (I_{\text{peak}} - I_{\infty}) \cdot \exp\left[\left(\frac{-(t-t_0)}{\tau}\right)^{\beta}\right] \quad (1)$$

where τ is the decay time, t_0 is the time at which the presynaptic spike finishes, β is the correction factor, which is 1, I_{peak} is the amplitude of the EPSC, and I_{∞} is the final value of the decay current.

Here, τ is estimated to be ~ 54 ms for the contact-electrification-activated synaptic transistor, which means that the feature time of ion migration is ~ 54 ms.

Supplementary Note 4

Sensory memory : The decay time τ is related to the detailed process of synaptic activation, ranging from tens of milliseconds to several minutes in time scale.[1] In psychology, "the multistore model" of brain memory proposed by Atkinson and Shiffrin[2] is widely accepted as illustrated in Figure S13a. The model consists of three store concepts, which are namely a sensory memory (SM), a short-term store/working memory (STM) and a long-term memory (LTM). Information from the external stimulation is stored for a very short period of time in the sensory register as a SM, and then selected information is transferred from STM (15-20 seconds) to a permanent LTM (few minutes to a couple of hours).[3,4] In addition, STM can become LTM through a process of rehearsal, and that the probability of transfer to LTM increases with rehearsal repetition.

A survey of the decay time of the previous literature has been listed in Supplementary Table 1 and Figure S13b. Compared with other reports, the decay time of our device triggered by single pulse is in the range of SM stage. This value can fluctuate according to the amplitude, duration time, and pulses number of the external stimulation. It has the potential to transform from SM to STM model. Further engineering on the architecture of the synaptic transistor or develop proper training methods is probably to realize the LTM model.

Supplementary Note 5

Energy dissipation: As a three terminal synaptic device, the total energy consumption per spike should be considered from both drain-source terminal and gate-source terminal, which can be evaluated by the sum of the resistive energy ($E_{\mathrm{resistive}}$) and the capacitive energy ($E_{\mathrm{capacitive}}$).

$$E_{\text{total}} = E_{\text{resistive}} + E_{\text{capacitive}} \quad (2)$$

The $E_{\text{resistive}}$ is determined by the channel current and drain voltage, which can be calculated from:

$$E_{\text{resistive}} = V_D \times I_{\text{peak}} \times t \quad (3)$$

Where I_{peak} is the peak current, V_D is the source-drain voltage, and t is the presynaptic-spike duration, respectively. To evaluate the $E_{\text{resistive}}$, the peak current of EPSC is measured to be ~119 pA at V_D = 1 mV with spike duration of 0.1 s, as shown in **Figure S15b**. Accordingly, the resistive energy of single spike event is estimated to be ~11.9 fJ.

The $E_{\text{capacitive}}$ determined by the gate voltage and gate dielectrics is evaluated by the equation:

$$E_{\text{capacitive}} = \frac{1}{2} C_{\text{eff}} V_{\text{ps}}^2 = \frac{(I_{\text{peak}} t)^2}{2C_{\text{eff}}} = \frac{(Q_c)^2}{2C_{\text{eff}}} \quad (4)$$

where C_{eff} and V_{ps} are the effective capacitance and the externally supplied presynaptic spike voltage, respectively.

To estimate the capacitive charging energy per spike event, the C_{eff} of the ion gel is measured in Figure S4d. The effective capacitance per unit area is about 3 μF·cm^{-2} at f = 1 Hz. By multiplying the measured capacitance (3 μF·cm^{-2}) and device area (50 μm^2), the C_{eff} is calculated to be 1.5 pF = 3 μF·cm^{-2} × 50 μm^2. The calculated capacitive charging energy is 47.2 pJ. Corresponding parameter values used in the calculation are shown in Supplementary Table 2.

For the traditional artificial synaptic transistor, the presynaptic spike is applied with the electrical pulse, which should be counted to evaluate the total energy consumption. From the above calculation results, the capacitive energy is much higher than the resistive energy and can't be neglected.

In contrast, for CE-activated artificial afferent neuron, the electrical energy of capacitive term is replaced by the mechanical energy of the action spike, which is a self-powered process without any applied external electric voltage (i.e., the externally supplied presynaptic spike voltage V_{ps} = 0, from which the $E_{\text{capacitive}}$ is evaluated to be 0). Therefore, by using the

triboelectric potential to power the synaptic transistor can remove the capacitive energy and greatly reduce the total energy consumption. Notably, the back-end processing circuit and flash LED circuits are part of demonstrations instead of the necessary component of the CE-activated artificial afferent. Their energy dissipations are excluded for evaluation of the ultralow power consumption of CE-activated artificial afferent (Supplementary Table 3).

Supplementary Note 6

Pressure-sensing mode: The contact-electrification-activated artificial afferents in pressure-sensing mode are designed with a three-point bending architecture[5-8], which is similar to the TENG geometry (Al/PTFE/Al) in contact-separation mode. Additionally, an acrylic spacer is used to support the top Al electrode. The height of the acrylic spacer (*h*) is 4 mm, and the valid length (*l*) of the PTFE is 25 mm. The deflection is defined according to the moving distance (*D*) of the top Al electrode upon center loading.

The contact-electrification-activated artificial afferents in the three-point bending architecture is used to monitor the external pressure. The applied pressures lead to the displacements between the top Al electrode and PTFE layer with a nonlinear relationship (Figure S19c). The external-pressure-induced displacement is equivalent to the contact-separation process of the TENG. The applied pressure at the top Al film induces electron transfer from the presynaptic terminal to the Al electrode, resulting in a positive triboelectric potential coupled to the MoS_2 synaptic transistor and modulating the channel conductance and the corresponding postsynaptic current. The peak value of the triggered EPSC increases from 0.1 to 1.8 nA as the applied external pressure increases from 6 to 130 kPa (Figure S19d). The sensitivity is divided into two regions: 12.3 kPa^{-1} and 3.7 kPa^{-1} for region *I* and region *II*, respectively (Figure S19e).

Supplementary Note 7

Micrometer-/nanometer-scale applications of CE-activated artificial afferent: To develop the contact-electrification-activated afferent in lower dimension (e.g., micrometer scale), active matrix design of flexible transistor array is an optimal option, which has been well demonstrated based on inorganic,[9] organic,[10,11] and low dimensional active nanomaterials[12,13] in last decades. Inspired by previous research, contact-electrification-activated transistor array can also be fabricated by designing proper transistor structure. For example, with a top gate design, a flexible tribotronic transistor array (10 × 10) with each sensing pixel scaled down to the size of 500 × 500 μm^2 has been demonstrated as an active tactile sensor.[14] Besides, by utilizing ion gel as dielectrics, a direct-contact-mode tribotronic planar graphene transistor array are also successfully fabricated and further scaled down to the size of 50 × 300 μm^2.[15] The critical issue of scaling-down the contact-electrification-activated transistor is to maintain efficient gating properties with the triboelectrification component at a smaller size, which generally inevitably shows a decreased input to transistor. Therefore, further improving the output of triboelectrification part and developing high-κ or mechanically robust electrolyte dielectrics are the key points to develop sophisticated contact-electrification-activated afferent in micrometer scale.

For the nanometer scale applications, atomic force microscopy (AFM) and Kelvin probe force microscopy (KPFM) are efficient means to investigating the electron transfer, surface potential, and triboelectric charge decay in nanometer-scale contact electrification.[16] A nanoscale triboelectrification-gated transistor has recently been studied based on contact-mode AFM and KPFM.[17-19] Charge carrier density in transistor channel can be modulated by the AFM (or KPFM) tip at a nanoscale. The fundamental researches on the influences of contact force, scan speed, contact cycles, contact region and charge diffusion are beneficial for developing the contact-electrification activated transistor into pressure or other types of sensors in nanometer scale. Furthermore, a controllable voltage can be applied to the AFM tip for nanoscale triboelectrification to serve as a rewritable floating gate, which offers a

possible way to developing a nanoscale neuromorphic device with in-memory computing functions.[20] The integration of nanometer scale device also requires paired nano-fabrication or nano-electromechanics techniques.

Supplementary Note 8

Possible solutions to alleviating the susceptible issues of ion gel to humidity/temperature variations: As is known, most existing ion gels are sensitive to humidity because the ion liquids can absorb moisture from air in a high-humidity atmosphere, which may result in the swelling or performance degradation of the ion gels and influence the FET properties.[21-23] For the temperature variation, especially when the temperature is below the glass transition temperature, the polymer chain and ion liquid may be frozen, and the ions contained in the ion gel can only slowly move in response to external electric field. As a result, the ionic conductivity will be decreased and the electrical double layer cannot form at the ion gel/electrode interface, leading to decreased effective capacitance and degraded electrical performances.[24]

Therefore, several effective strategies have been demonstrated to alleviate the susceptibility of ion gel to surrounding moisture/temperature variations. Firstly, encapsulation of the ion-gel devices with elastomer coating can isolate the molecular exchange between ionic liquid/gel and external environment so as to reduce the impact of external environment changes.[25,26] Secondly, introducing highly hydratable salt to be composited with ion gel can readily maintain the water absorption/retention capacity due to the intrinsically stable hygroscopicity of the concentrated salts.[27] Thirdly, elaborate selection or synthesis of hydrophobic and chemically stable ionic liquid paired with relevant processing conditions and proper solvent/gelation polymers with excellent extreme-temperature tolerance can effectively alleviate the humidity/temperature susceptible issues.[24] Besides, according to demanded experiment process, conducting the characterization on ion-gel devices in an inert atmosphere

environment or vacuum measurement chamber can readily avoid long-term air exposure and weaken the influence of humidity/temperature.[28]

From another point of view, thanks to the response of ionic gel/liquid to humidity/temperature change, versatile humidity sensors, thermal sensing and thermoelectric gating applications have been demonstrated,[29] e.g., spiking humidity detection,[30] polyelectrolyte interlayer ultra-sensitive humidity sensing,[31] external heat-gated transistors by some electrolytes with high Seebeck coefficient,[32] gate-tunable thermoelectric devices,[33] etc. These versatile applications are possible to be combined with the CE-activation sensing and extended to multimodal humidity/thermal/mechano-sensation.

Supplementary Figures

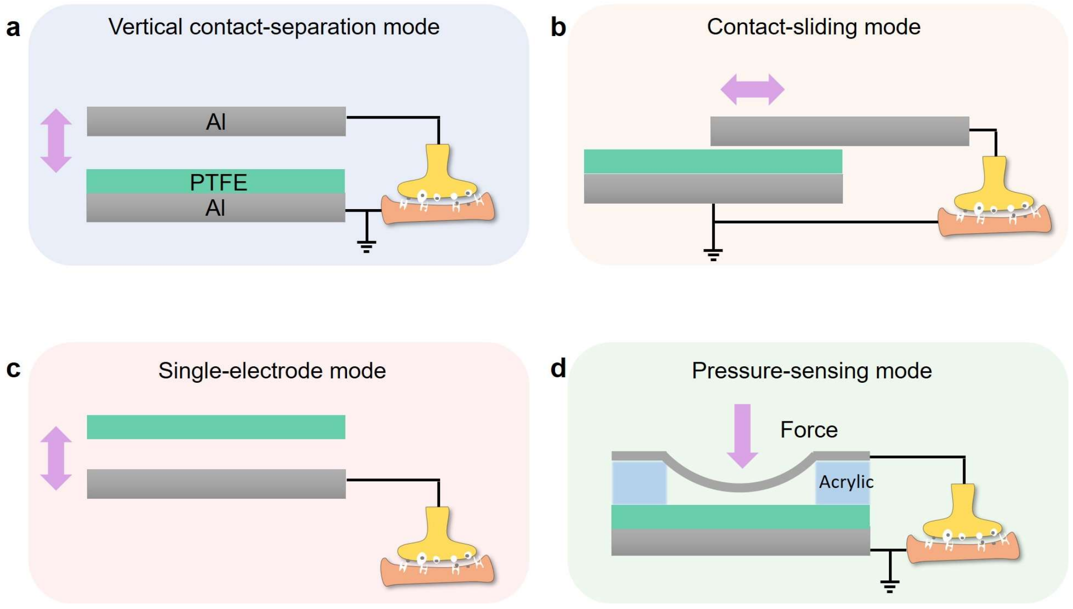

Supplementary Figure 1 | Schematic illustration of different working modes for the contact-electrification-activated artificial afferents. a, Vertical contact-separation mode. **b**, Contact-sliding mode. **c**, Single-electrode mode. **d**, Pressure-sensing mode.

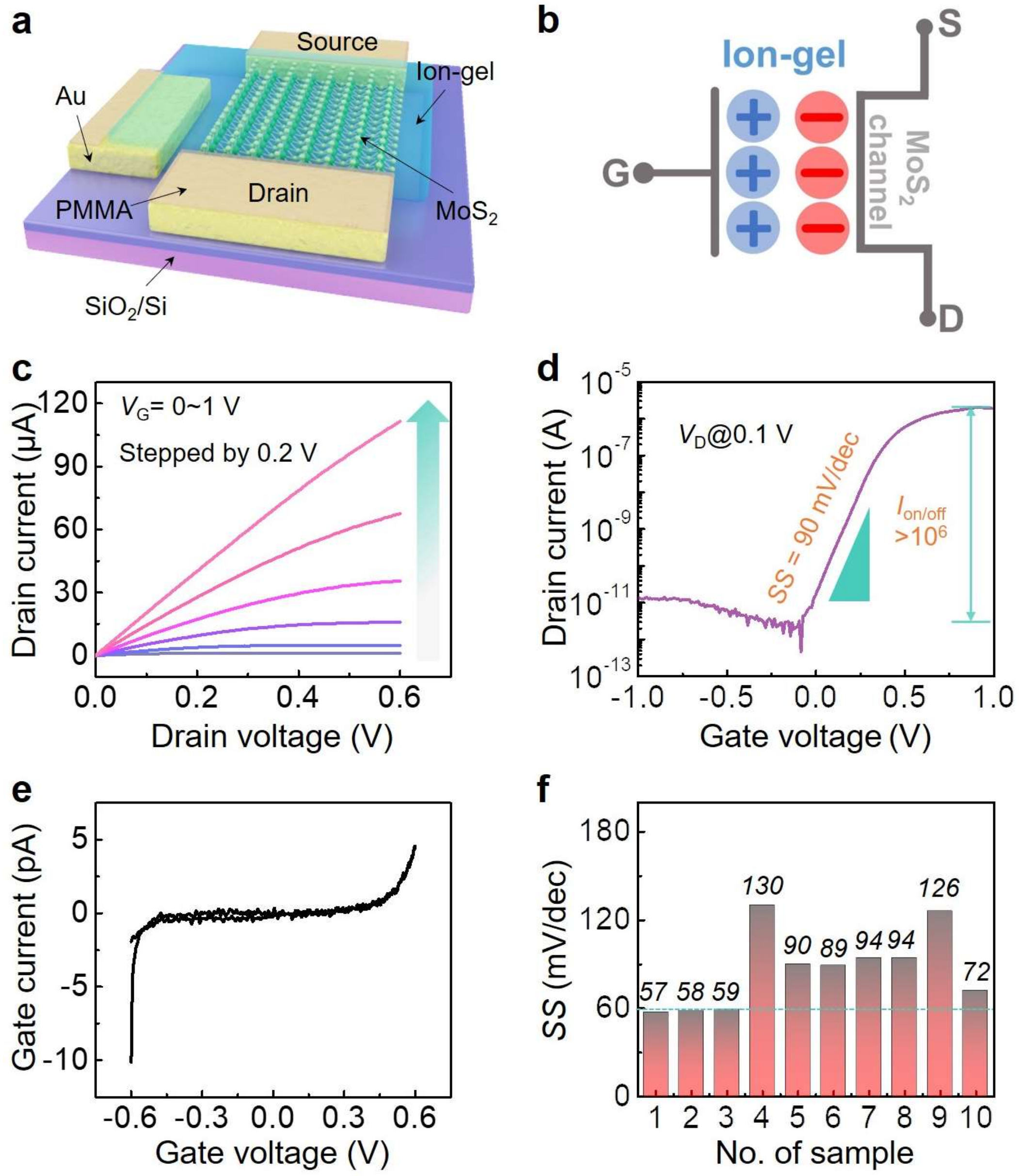

Supplementary Figure 2 | Electrical characterization of the MoS_2 FET. a, Schematic illustration of the MoS_2 FET on a SiO_2/Si wafer. It includes a planar gate, source and drain electrodes, a MoS_2 channel and an ion gel dielectric layer. The patterned PMMA (150 nm) on the source-drain electrode ensures a low leakage current. **b**, Circuit diagram of the MoS_2 FET. Upon application of the gate voltage, the EDLs are induced at the interfaces of the gate/ion gel and ion gel/channel, which can induce a high density of charge carriers in the channel and highly effectively modulate the output current. **c**, The typical output performances (I_D-V_D) of the MoS_2 FET under V_D sweeping (0~0.6 V) at different V_G values (0~1 V) with a step of 0.2

V. The output curve shows an increase in MoS_2 channel conductance with increasing V_G. Good pinch-off characteristics and excellent linear characteristics are observed in the high V_D and low V_D regions, which are attributed to the strong gate modulation at low V_G (<1 V) according to the formation of an EDL in the ion gel. Low-voltage operation is highly desired to meet the critical requirement of low power consumption in synaptic devices. **d**, The corresponding transfer curve in the logarithmic scale of the MoS_2 FET at a V_D of 0.1 V with the V_G swept from -1 to 1 V. The current on/off ratio exceeds six orders of magnitude. **e**, The gate leakage current (I_G) of the MoS_2 synaptic transistor is ultralow (at the level of several pA) due to the encapsulation of the PMMA layer. **f**, Statistics of subthreshold swing (*SS*) for ion-gel-gated MoS_2 transistors. The average *SS* is estimated to be 87 mV/dec. Thereinto, three of the prepared transistors show *SS* smaller than 60 mV/dec, which may be attributed to the ultrahigh capacitance of the formed EDLs during operation of ion-gel-gated MoS_2 FET. The ultrahigh capacitance greatly decreases the V_G, but still leads to relatively high I_D. According to the equation of $SS = dV_G/log_{10}I_D$, the SS in some devices may be deviated and underestimated to be lower than 60 mV/dec (i.e., the thermionic limitation).

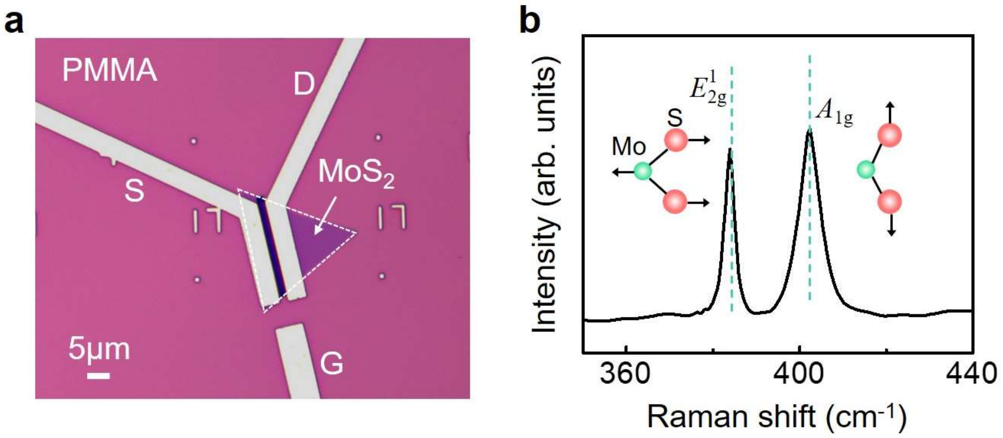

Supplementary Figure 3 | Optical properties of MoS_2. a, Optical microscope image of the MoS_2 synaptic transistor before patterning of the ion gel. The triangular MoS_2 flake is grown on silicon wafers. The channel length and width are ~ 2 and 10 μm, respectively. **b**, The

Raman spectrum of the synthesized MoS_2. Two major peaks are located at ~384 and ~409 cm^{-1}, corresponding to the in-plane E_{2g}^{1} mode and the out-of-plane A_{1g} mode, respectively. The E_{2g}^{1} mode vibration involves the in-plane opposing motions of sulfur and molybdenum atoms, while the A_{1g} mode is related to the out-of-plane relative motions of sulfur atoms.

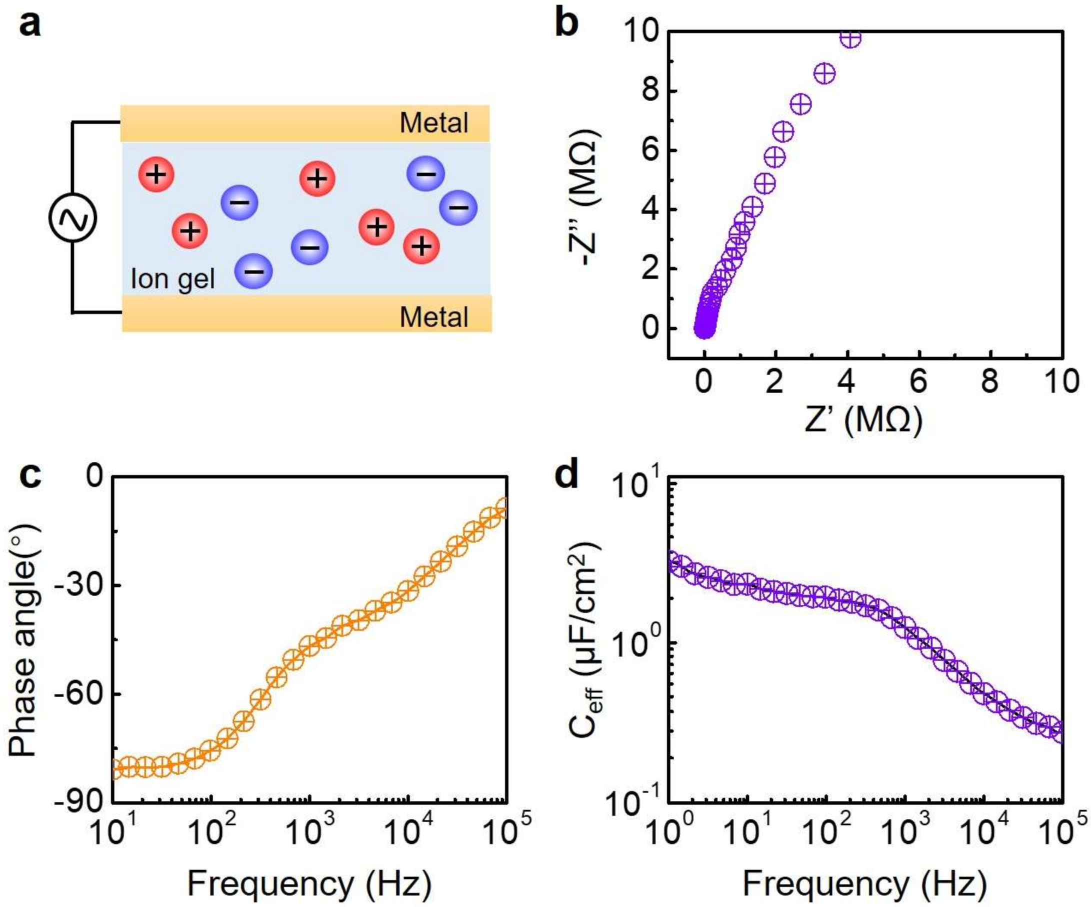

Supplementary Figure 4 | Impedance analysis of ion gel dielectrics. a, Schematic illustration of the ion gel dielectrics in a typical metal-insulator-metal structure. Ion gel can be used as a dielectric layer according to its ionic conducing yet electronic insulating properties. **b**, The real and imaginary parts of the ion gel impedance in Nyquist plots. An inclined spur in the low-frequency region can be observed, which indicates an ideal capacitive behavior of the EDL layer. **c**, The phase angle curves of the ion gel as a function of the frequency. The phase angle is -90° for an ideal capacitor and 0° for an ideal resistor. In the low frequency region, the phase angle $\theta < -45°$ indicates that the ion gel follows capacitive behavior rather than a

resistive one, and that the effective capacitive is high. The results are attributed to the formation of EDLs. In the high frequency region, the phase angle is ＞-45°, which indicates that the ion gel tends to exhibit more resistive behavior due to the limited response of ion migration. **d**, The effective capacitance (C_{eff}) of the ion gel *vs.* the applied AC frequency. The C_{eff} is ultrahigh (~10 μF·cm^{-2}) at low frequency (1 Hz) and still higher than 1 μF·cm^{-2} at 2000 Hz, representing stable capacitive performance for the transistor operation.

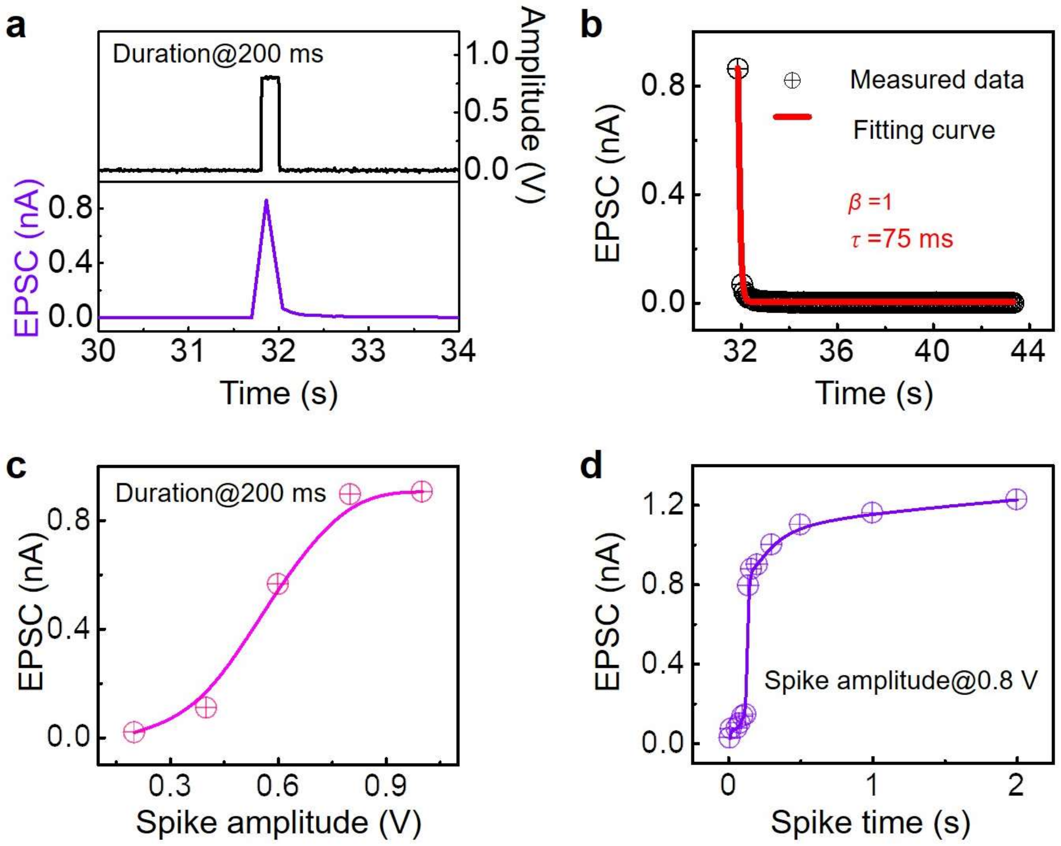

Supplementary Figure 5 | Synaptic characteristics of the MoS_2 transistor under applied gate spikes. a, Typical EPSC behavior with a peak current of 0.86 nA is observed under a gate pulse of 0.8 V (duration of ~200 ms). **b**, The decay time is estimated to be 75 ms according to the fitting curve of the EPSC decay (0.87 nA) following the equation in the Supplementary Note. **c**, EPSC *vs.* spike amplitude. The EPSC shows an increment from 0.02 to 0.9 nA according to the increased gate voltage spike from 0.2 to 1 V (duration of ~200 ms). **d**, EPSC *vs.* spike time. The peak value of the EPSC shows a steep increment (spike duration from 0 to 0.3 s) with a subsequent saturation tendency (0.3 to 2 s). This result is attributed to

the fact that the ions contributing to the EDLs gradually become saturated under longer periods of spike gating.

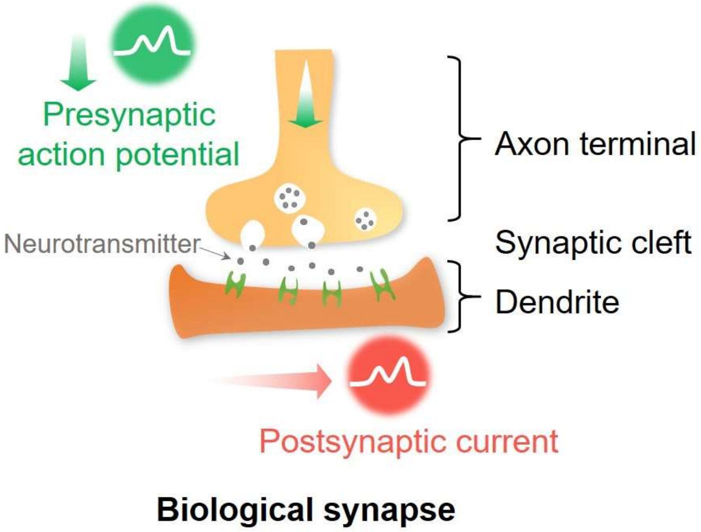

Supplementary Figure 6 | Biological synapse. A typical biological synapse is composed of a presynaptic membrane (the axon terminal of the preneuron), a synaptic cleft and a postsynaptic membrane (the axon, dendrite or soma cell of the postneuron). The action potential transmission of the biological synapses occurs as follows. First, the nerve action potential (internal electric signal) is initiated by an external mechanical signal captured by the sensory neuron, *i.e.,* the signal perception. The action potential propagates along the nerve fiber and intrudes into the presynaptic terminal to activate the voltage-driven calcium (Ca^{2+}) channels on the presynaptic membrane. According to the increase in calcium permeability, the neurotransmitters wrapped in synaptic vesicles are immediately released into the synaptic cleft driven by the calcium ions. The neurotransmitters diffuse across the synaptic cleft and bind to protein receptors on the postsynaptic membrane, inducing changes in sodium (Na^{+}) and potassium (K^{+}) permeability. The flow of sodium and potassium leads to a potential depolarization of the postsynaptic membrane and induces the excitatory postsynaptic current, *i.e.,* EPSC, which leads to the initiation of another action potential in the subsequent nerve cell.

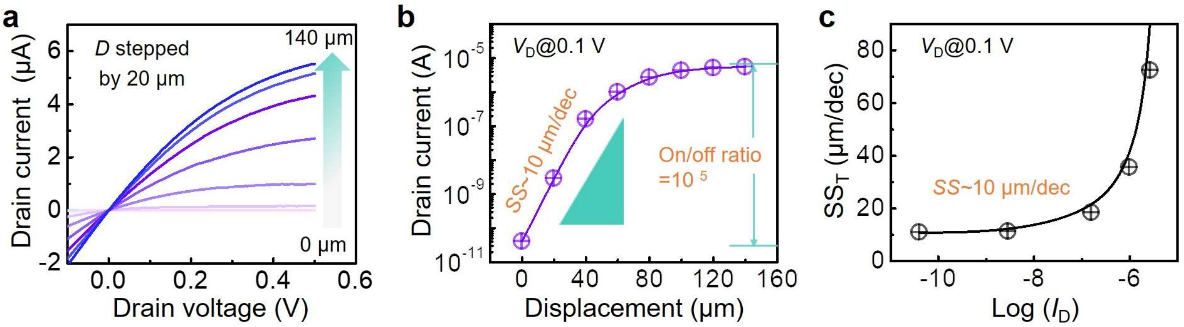

Supplementary Figure 7 | Electrical characterization of the tribo-iontronic MoS_2 FET. In the contact-electrification gated devices, the gate voltage is replaced with the triboelectric potential induced by the displacement of the TENG. The original stepped and swept gate voltages for the output and transfer characteristics are all replaced with the displacement. **a**, The output performance (I_D *vs.* V_D) of the tribo-iontronic MoS_2 transistor under different displacements (*D*). The drain current increases with increasing displacement. **b**, The extracted transfer curve (I_D *vs.* *D*) of the tribo-iontronic MoS_2 transistor. The drain current increased from 40 pA to 5.5 μA as the displacement changed from 0 to 140 μm at a V_D of 0.1 V. The current I_D shows a linear increment at a small friction distance *D* and then becomes saturated under a large friction distance with a large current on/off ratio exceeding 10^5. The above results demonstrate that the triboelectric potential originating from the displacement of the TENG can effectively drive the transistor. **c**, As figure of merits to evaluate the energy consumption of the tribo-iontronic transistor, an analogue to the subthreshold swing (*i.e.*, tribotronic subthreshold swing, SS_T) is plotted versus log (I_D), which is extracted from the transfer curve of the tribo-iontronic MoS_2 synaptic transistor. The SS_T is estimated to be as small as 10 μm/dec and remains below 40 μm/dec under a current variation of approximately four orders of magnitude. The excellent modulation capacity of the TENG displacement presumes artificial synapses with excellent performance.

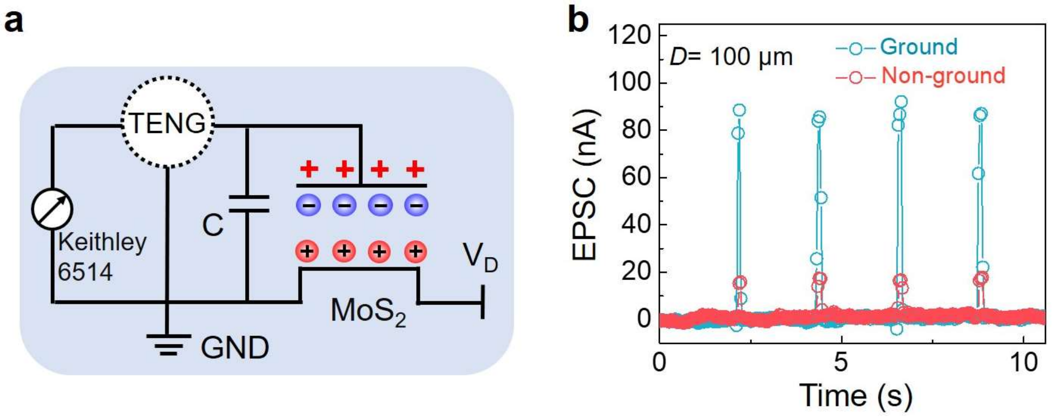

Supplementary Figure 8 | Grounding reference investigation. a, Circuit diagram for the operation of CE-activated artificial afferents. The TENG self-activation component has the common grounding reference with the source electrode. The equivalent gate voltage produced by the TENG component is monitored by an electrometer (Keithley 6514) during test in real-time. **b**, EPSCs according to the external displacement of 100 μm with/without common grounding reference. The common grounding reference between source electrode and TENG electrode is critical to the efficient self-activation process. As shown in Figure S8b, without the common grounding reference, the EPSC according to the external displacement of 100 μm is less than 20 nA. In contrast, with the common grounding reference, the EPSC response is significantly increased to 90 nA, demonstrating higher activation efficacy for better mechanosensation applications.

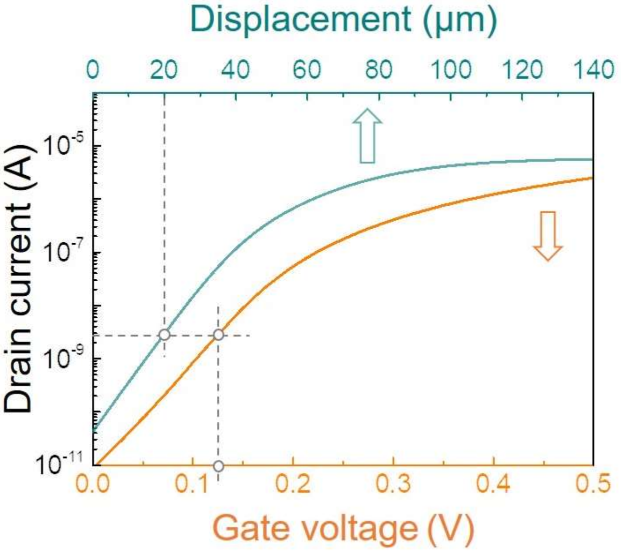

Supplementary Figure 9 | The transfer curve of MoS_2 transistor and corresponding tribotronic transfer curve to extract the equivalent V_G. At a displacement of 20 μm, the I_D is 2.9×10^{-9} A. To achieve the same I_D, the applied V_G is ~0.125 V, i.e., the equivalent V_G at a TENG displacement of 20 μm is 0.125 V.

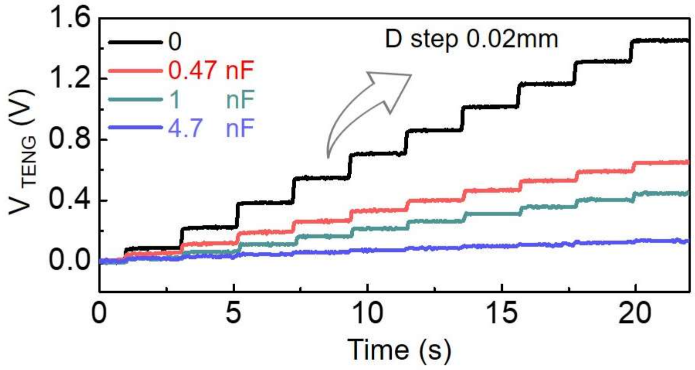

Supplementary Figure 10 | Charging curves of TENG with different capacitors (0, 0.47, 1, and 4.7 nF) in parallel.

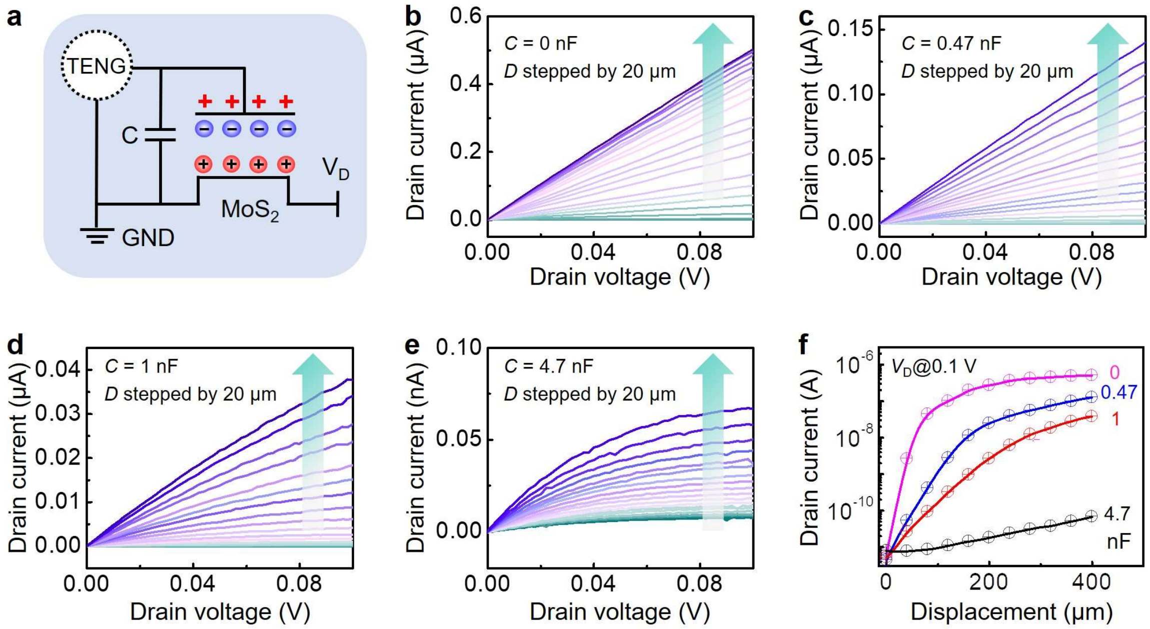

Supplementary Figure 11 | Investigations on connected capacitors. a, The equivalent circuit diagram of the contact-electrification-activated MoS_2 synaptic transistor connected with a capacitor in parallel. **b-e,** The output curves of the tribo-iontronic MoS_2 synaptic transistor connected with different capacitors (0, 0.47, 1, and 4.7 nF) in parallel. **f**, The extracted transfer curves from (b-e).

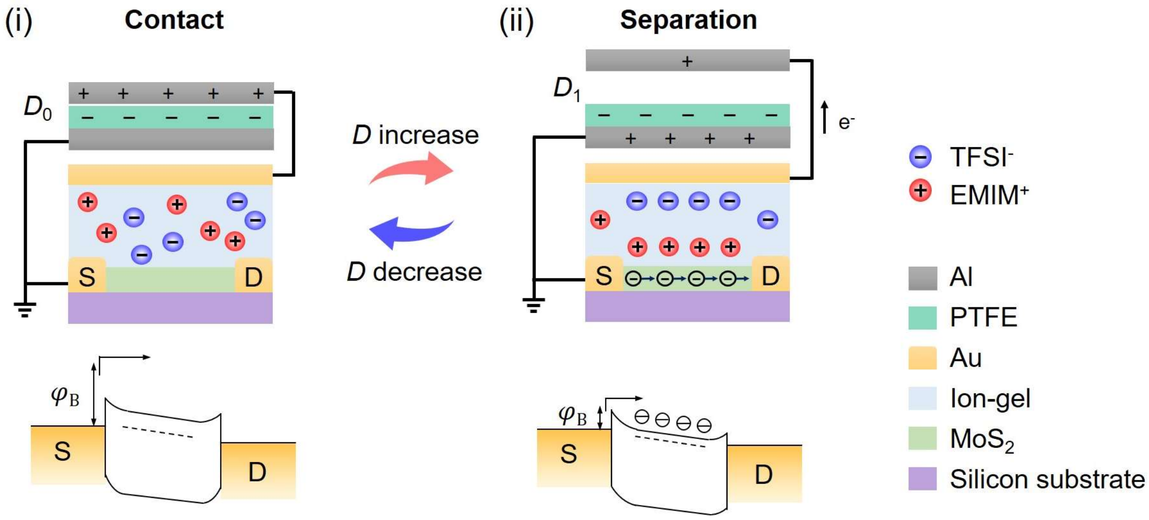

Supplementary Figure 12 | Schematic illustrations of the working mechanism and the corresponding energy band diagram of the contact-electrification-activated MoS_2 synaptic transistor. We take the contact-separation mode as an example. (i) The initial state of the tribo-iontronic MoS_2 synaptic transistor. The cations and anions are distributed randomly in the ion gel when the frictional layers of the TENG are in contact with each other (D=D_0). (ii) At a separation distance D_1, the cations and anions migrate to form the EDLs, accumulating in the MoS_2 channel.

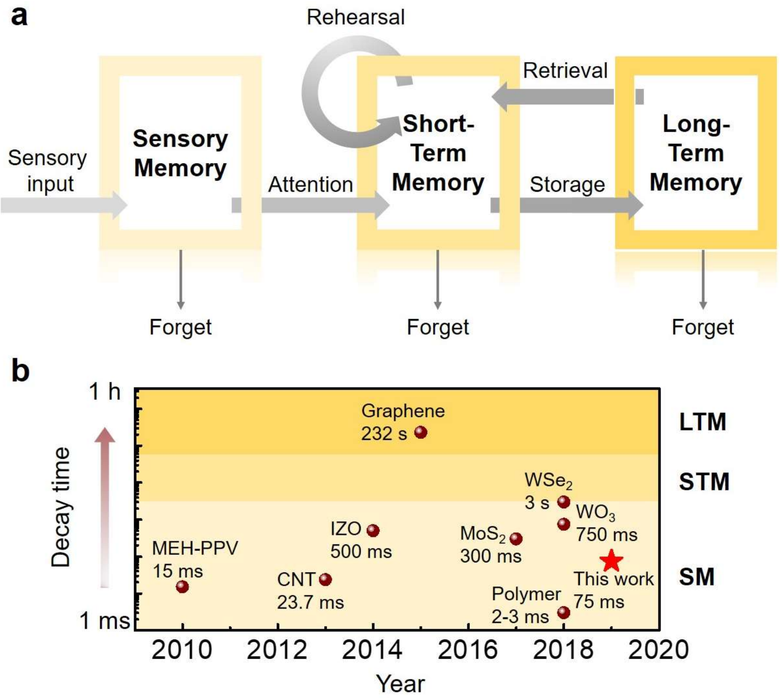

Supplementary Figure 13 | Sensory memory. **a**, Multistore model of brain memory—Atkinson & Shiffrin. **b**, Comparison of the decay time of the reported synaptic transistor in previous literatures.

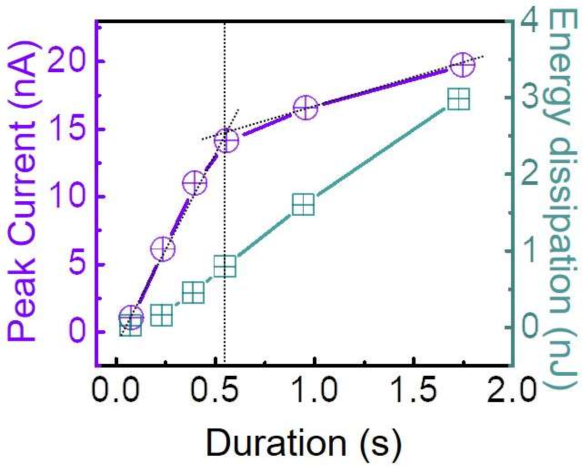

Supplementary Figure 14 | The peak current of the EPSC vs. duration time of the MoS_2 synaptic transistor in contact-separation mode. A linear increment (< 0.6 s) with a subsequent saturation tendency (0.6 ~1.75 s) is shown.

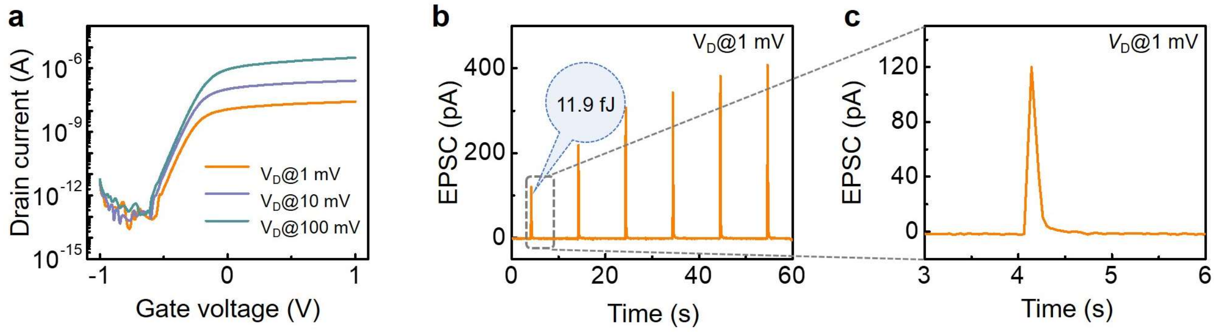

Supplementary Figure 15 | Evaluation of the energy dissipation at 11.9 fJ at V_D = 1 V. a, Transfer curves of the synaptic transistor under different V_Ds. **b**, The EPSC responses under different CS actions durations (D = 50 μm) when V_D=1 mV. **c**, The EPSC responses under a CS action duration (D = 50 μm, duration time is 0.1s).

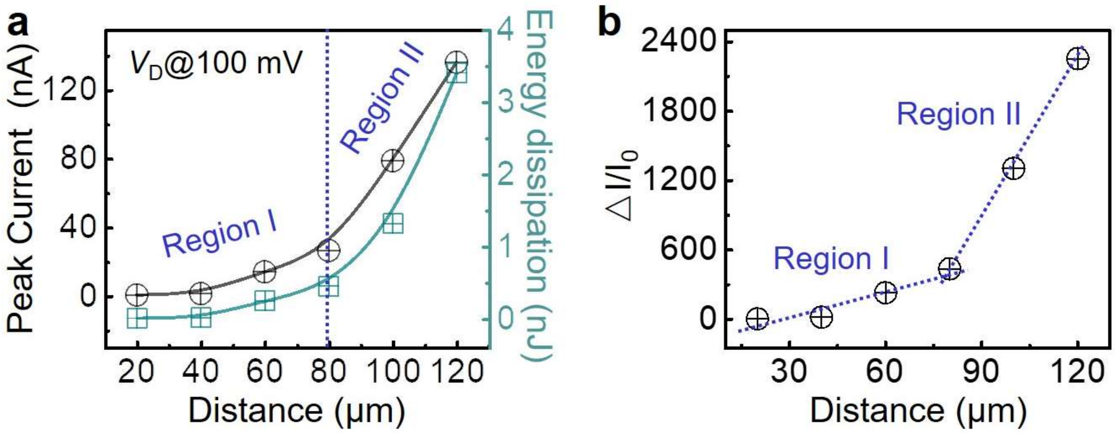

Supplementary Figure 16 | Peak current and $\triangle I/I_0$ vs. displacement. a, The EPSC peak current *vs.* displacement, which is divided into two regions (Region *I* and Region *II*) according to the variation tendency. **b**, The sensitivity of the contact-electrification-activated artificial afferents in contact-separation mode, defined as $(\triangle I/I_0)/D$, which is 7 μm^{-1} and 45 μm^{-1} for region *I* and region *II*, respectively.

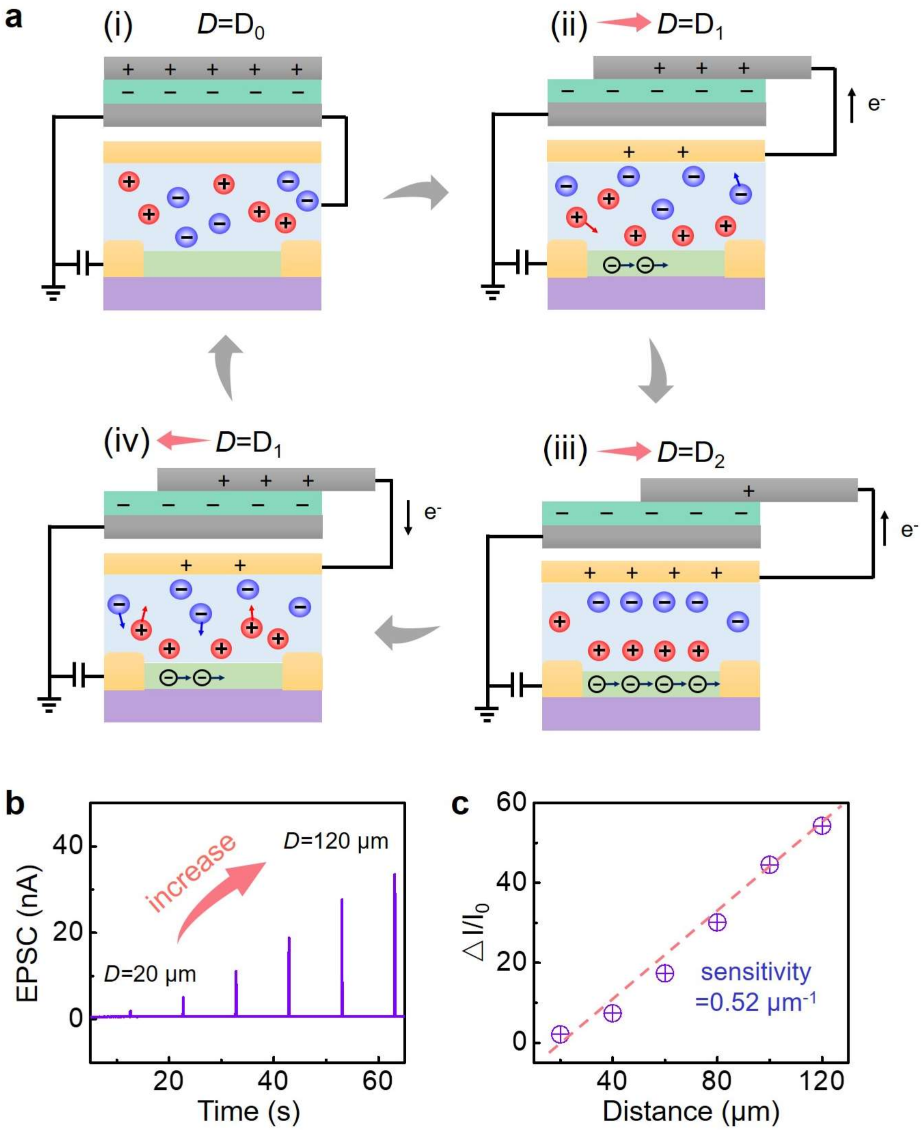

Supplementary Figure 17 | Contact-sliding mode. a, The working mechanism of the tribo-iontronic MoS_2 synaptic transistor in contact-sliding mode. (i) The cations and anions are distributed randomly in the ion gel at the initial state (D_0). (ii) The cations and anions start to migrate following the sliding of the Al electrode (D_1). (iii) The cations and anions further migrate to form EDLs with the continuous sliding of the Al layer to a larger distance (D_2). (iv) The cations and anions gradually diffuse back to the initial state during the sliding back of the Al electrode (D_2 to D_1). **b**, The EPSC at different displacements. **c**, The sensitivity of the contact-electrification-activated artificial afferents in sliding mode (0.52 μm^{-1}).

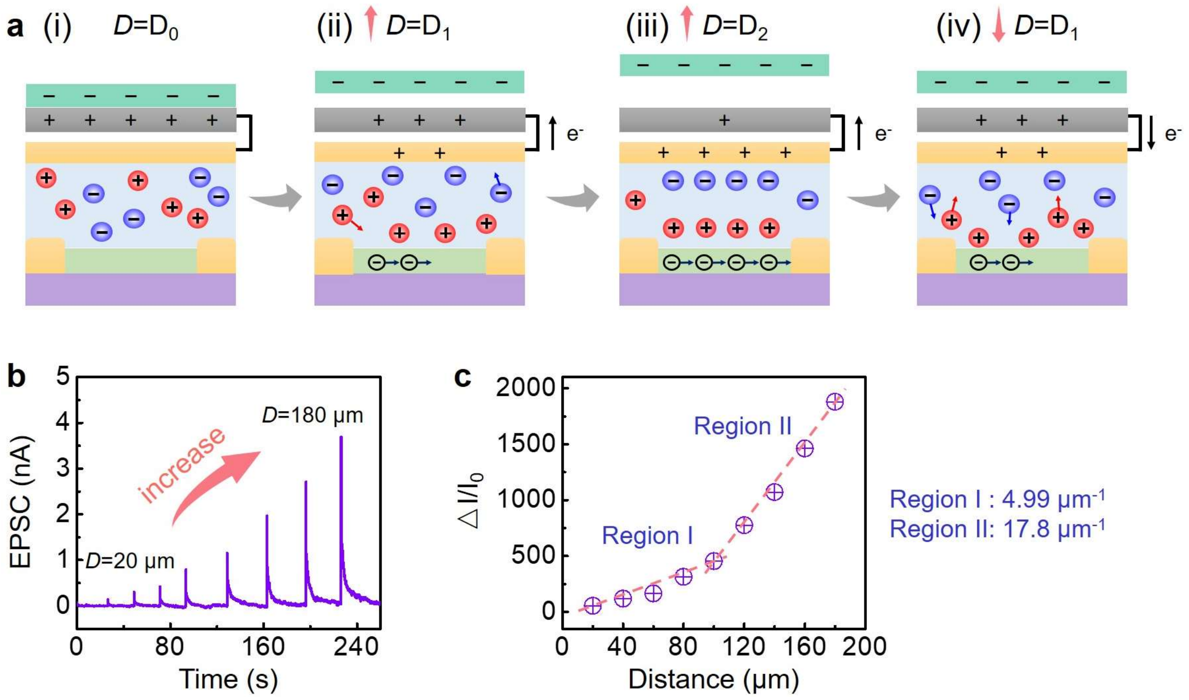

Supplementary Figure 18 | Single-electrode mode. a, The working mechanism of the tribo-iontronic MoS_2 synaptic transistor in single-electrode mode. Similar to the contact-separation mode, the displacement between the target object and sensing electrode leads to triboelectric potential coupled to the synaptic transistor, which induces the formation of EDLs and modulates the charge carrier density in the MoS_2 channel. **b**, The EPSC at different separation distances. **c**, The sensitivity of the contact-electrification-activated artificial afferents in single-electrode mode, which is 4.99 μm^{-1} and 17.8 μm^{-1} for region *I* and region *II*, respectively.

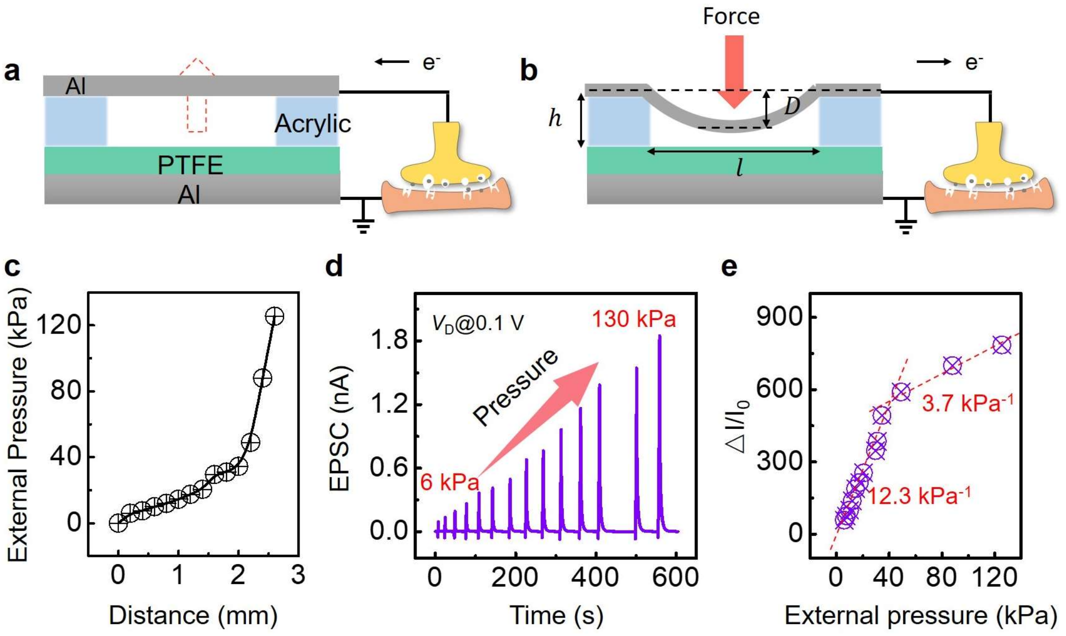

Supplementary Figure 19 | Pressure-sensing mode. a-b, The working mechanism of the tribo-iontronic MoS_2 synaptic transistor in pressure-sensing mode. **c**, The applied external pressure determines the displacement of the TENG. **d**, The EPSC under different pressures. **e**, The sensitivity of the contact-electrification-activated artificial afferents in pressure-sensing mode, defined as $(\triangle I/I_0)/P$, which is 12.3 kPa^{-1} and 3.7 kPa^{-1} for region *I* and region *II*, respectively.

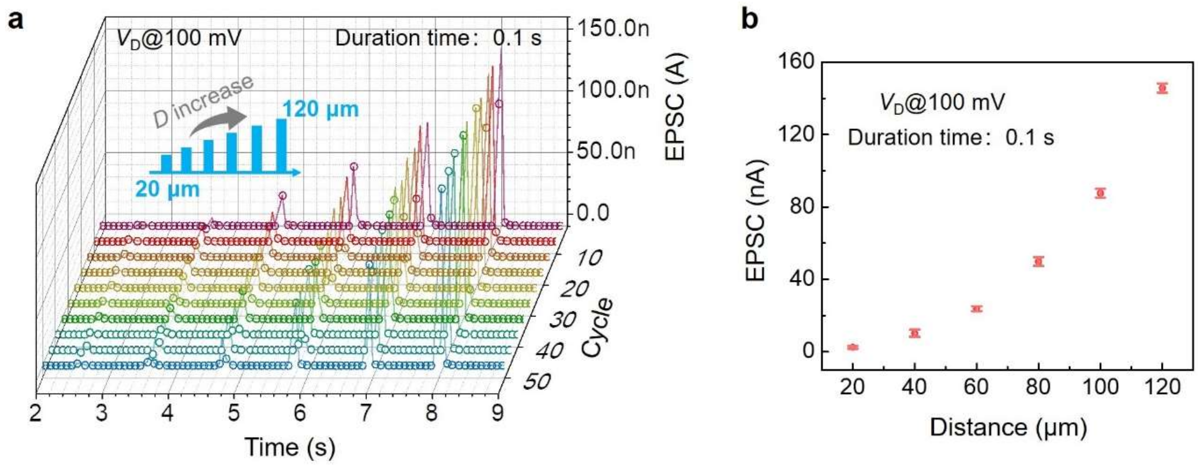

Supplementary Figure 20 | Reliability tests. a, Real-time EPSCs under six typical mechanical displacements. To demonstrate the reliability of CE-activated artificial afferents, six different mechanical displacements (20, 40, 60, 80, 100, and 120 μm) have been applied to

the device and corresponding EPSCs have been monitored in real time for 50 cycles. Under all the different mechanical displacements for 50 cycles, the measured EPSCs represent quite small deviations, indicating good reliability of the CE-activated artificial afferents. **b**, Cycle-to-cycle variations of the statistical EPSCs with error bars are observed to be smaller than 5%. The error bar is defined as the standard deviation.

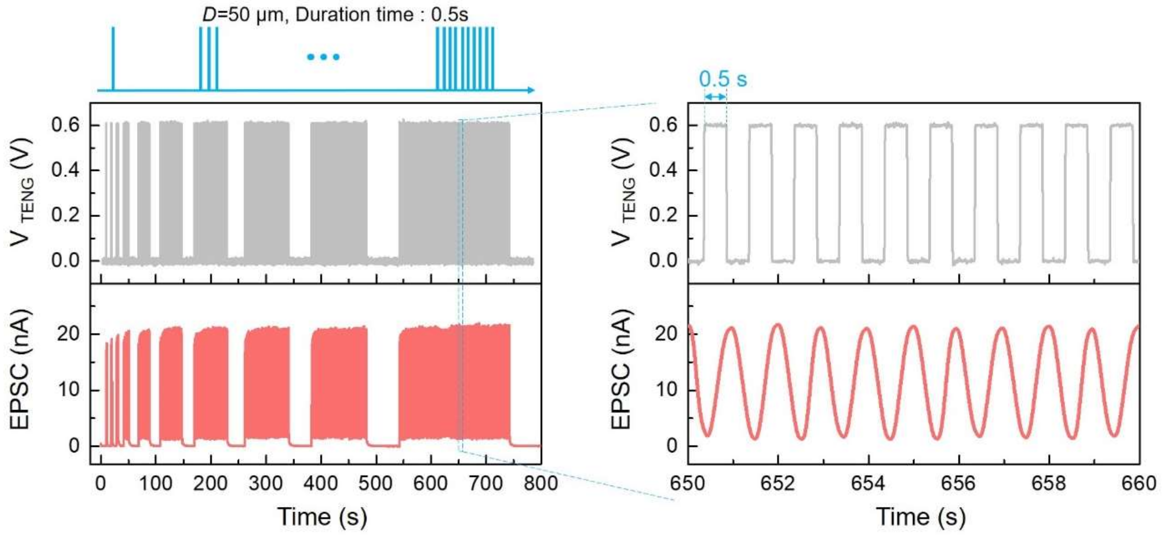

Supplementary Figure 21 | Durability test of The V_{TENG} and EPSC of the CE-activated artificial afferents. To demonstrate the robustness of the CE-activated artificial afferents, we have characterized the EPSCs upon applied mechanical displacements (*D* = 50 μm, duration at 0.5 s) for 1, 2, 4, 10, 20, 40, 60, 80, 100, and 200 cycles, respectively. Based on the stable outputs of TENG component at 0.6 V (i.e., equivalent gate voltages), the synaptic transistor delivers maintainable EPSCs at ~21 nA with no obvious base line drift, indicating the excellent durability of the CE-activated artificial afferent.

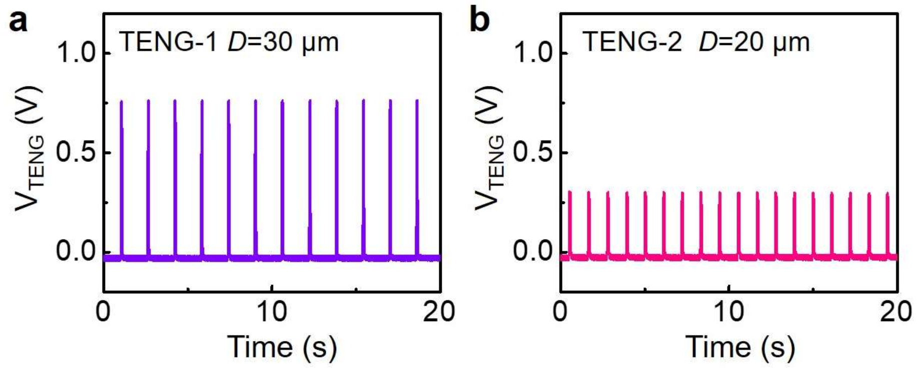

Supplementary Figure 22 | Output voltages of TENG-1 and TENG-2. The output voltages of TENG-1 and TENG-2 are 0.75 and 0.3 V at different displacements of 30 and 20 μm, respectively.

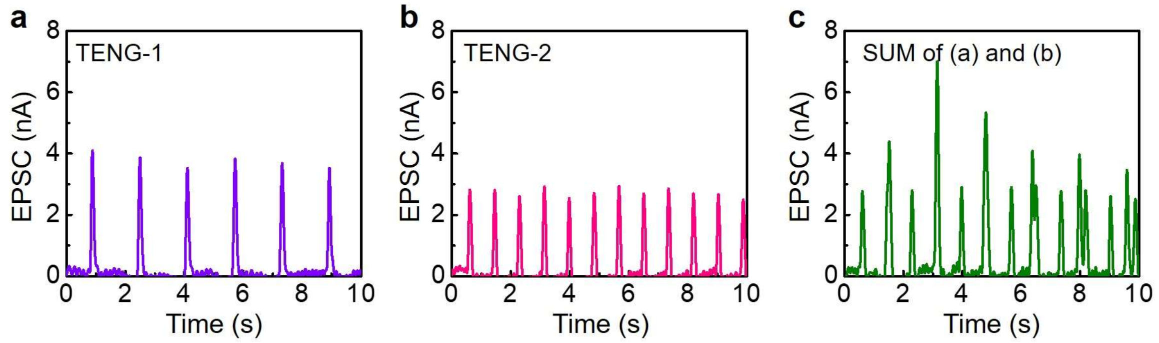

Supplementary Figure 23 | EPSC responses activated by TENG-1 and TENG-2. a, The EPSC responses of the synaptic transistor activated by TENG-1 (*D*=0.3 mm). **b**, The EPSC responses of the synaptic transistor activated by TENG-2 (*D*=0.2 mm). **c**, The mathematical combination of the EPSCs coordinates of TENG-1 and TENG-2.

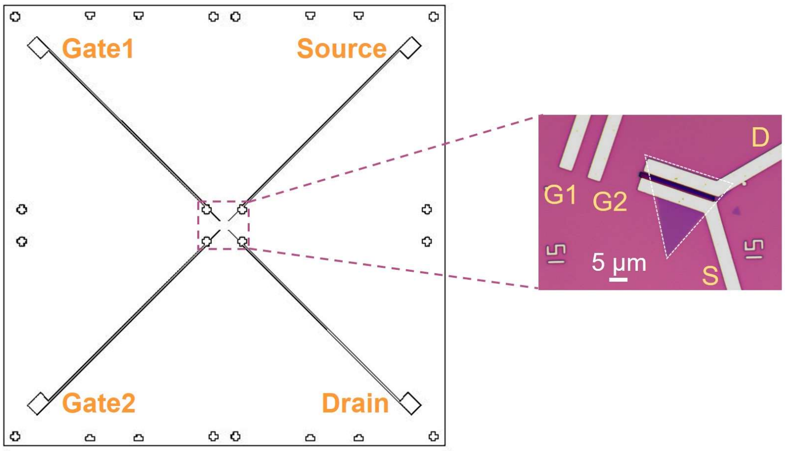

Supplementary Figure 24 | The pattern design (left) and the optical microscope image (right) of the contact-electrification-activated artificial afferent neuron.

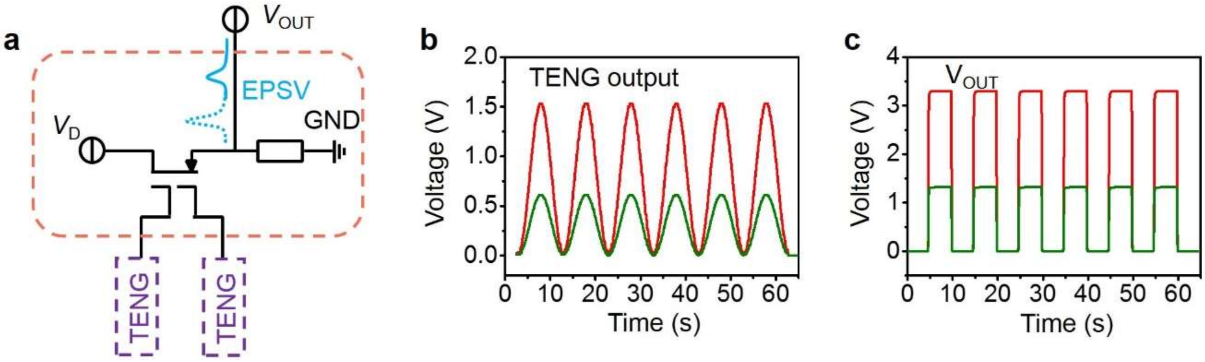

Supplementary Figure 25 | Circuit diagram for the demonstration of touch patterns recognition. **a**, The bleeder circuit for the EPSCs triggered from two TENGs upon touch patterns with spatiotemporal information, which is designed to achieve a more stable output trigger signal. **b**, The output voltage pulses of TENG-1 (2×2 cm^2) and TENG-2 (1.5×1.5 cm^2) under similar touch patterns. **c**, The output trigger voltages originating from TENG-1 and TENG-2, which are converted by the bleeder circuit. When the triggered voltage is > 2 V (or < 2 V), the signal can be acquired and recognized to induce a series of red (or green) lights to illuminate.

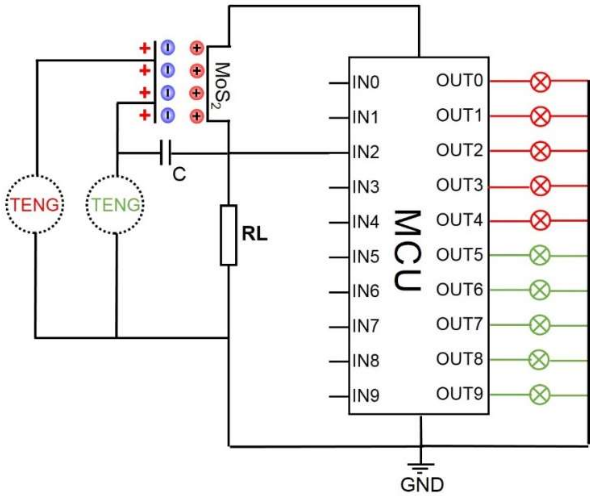

Supplementary Figure 26 | Circuit diagram of the spatiotemporal information recognition. After the touch patterns with spatiotemporal information are converted into electric signals through the contact-electrification-activated artificial afferent neuron, they are effectively acquired and recognized by the MCU to trigger the flash LEDs.

Supplementary Table 1 | The summarized decay time and energy dissipation of the reported artificial synapses or afferents based on FET devices in previous literature.

Year	Channel material	Dielectric layer	V_{DS}	Duration time	Decay time	Presynaptic Input voltage	Energy consumption/spike	Refs.
2010	MEH-PPV	$RbAg_4I_5$	N/A	N/A	**15 ms**	N/A	~10 pJ	34
2013	CNT	Hydrogen-doped PEG	0.1 V	1 ms	**23.7 ms**	5 V	~7.5 pJ	35
2014	IZO	Proton conductor	0.5 V	10 ms	**500 ms**	0.3 V	~45 pJ	36
2015	IZO	Chitosan	70 mV	10 ms	**N/A**	0.1 V	~3.9 pJ	37
2015	Graphene	AlO_X	0.1 V	10 ms	**232 s**	V_{tg}= 2 V<br>V_{bg}= 20 V	N/A	38
2016	ONW	[EMI][TFSI] in PS–PMMA–PS	20 mV	112 ms	**N/A**	-1 mV	~1.23 fJ	39
2017	MoS_2	PVA proton conductor	0.1 V	10 ms	**300 ms**	1 V	~23.6 pJ	40
2017	α-MoO_3	EMIM–TFSI	50 mV	1 ms	**N/A**	2.5 V	~9.6 pJ	41
2017	SWCNT	PFO-TP	0.1 V	10 ms	**N/A**	N/A	~1 pJ	42
2018	WSe_2	PEO: $LiClO_4$	0.5 V	100 μs	**~3 s**	N/A	~30 fJ	43
2018	MoS_2	(DEME-TFSI)	N/A	10.5 ms	**N/A**	N/A	~4.8 pJ	44
2018	WO_3	(DEME-TFSI)	0.3 V	70 ms	**750 ms**	0.6 V	36 pJ	45
2018	Polymer semiconductor	Ion gel	N/A	N/A	**2-3 ms**	N/A	N/A	46
2019	MoS_2	[EMIM][TFSI]	1 mV	0.1 s	**75 ms**	N/A	11.9 fJ	This work

Supplementary Table 2 | The parameter values used for the capacitive energy calculation at V_D = 1 mV.

Parameter	Value
Peak current of EPSC (I_{peak})	1.19×10^{-10} A
Spike duration (t)	0.1 s
Stored charge per synaptic event (Q_C)	1.19×10^{-11} C
Effective capacitance (C_{eff})	1.5×10^{-12} F
Capacitive energy $\frac{(Q_C)^2}{2C_{eff}}$	4.72×10^{-11} J

Supplementary Table 3 | The energy consumptions of the dynamic logic with CE-activated artificial afferent and external loads.

Unit	Energy consumption
CE-activated artificial afferent neuron	11.9 fW
Microcontroller unit (MCU)	75 mW
LED	2.2 μW

Supplementary References

1 Zucker, R. S. & Regehr, W. G. Short-term synaptic plasticity. *Annu. Rev. Physiol.* **64**, 355-405, (2002).

2 Atkinson, R. C. & Shiffrin, R. M. in *Psychology of Learning and Motivation* 89-195

(Academic Press, Place, Published 1968).
3 Norah Hadi, A. Wish you were here: A psychological analysis using atkinson-shiffrin memory mode. *Journal of Literature and Art Studies* **7**, 521-527, (2017).
4 Ohno, T. et al. Short-term plasticity and long-term potentiation mimicked in single inorganic synapses. *Nature Mater.* **10**, 591-595, (2011).
5 Kim, J. H., Sun, Q. & Seo, S. Pressure dependent current-controllable devices based on organic thin film transistors by soft-contact lamination. *Org. Electron.* **11**, 964-968, (2010).
6 Sun, Q., Kim, J. H. & Seo, S. External pressure responsive device based on tunable organic inverter using soft contact lamination. *Org. Electron.* **14**, 2401-2405, (2013).
7 Mohyeddin, A. & Fereidoon, A. An analytical solution for the large deflection problem of Timoshenko beams under three-point bending. *Int. J. Mechan. Sci.* **78**, 135-139, (2014).
8 Li, D. K. & Li, X. F. Large deflection and rotation of Timoshenko beams with frictional end supports under three-point bending. *C. R. Mecanique* **344**, 556-568, (2016).
9 Rogers, J. A., Someya, T. & Huang, Y. Materials and mechanics for stretchable electronics. *Science* **327**, 1603-1607, (2010).
10 Sekitani, T. et al. A rubberlike stretchable active matrix using elastic conductors. *Science* **321**, 1468-1472, (2008).
11 Takei, K. et al. Nanowire active-matrix circuitry for low-voltage macroscale artificial skin. *Nature Mater.* **9**, 821-826, (2010).
12 Wang, C. et al. User-interactive electronic skin for instantaneous pressure visualization. *Nature Mater.* **12**, 899-904, (2013).
13 Wang, S. et al. Skin electronics from scalable fabrication of an intrinsically stretchable transistor array. *Nature* **555**, 83-88, (2018).
14 Yang, Z. W. et al. Tribotronic transistor array as an active tactile sensing system. *ACS Nano* **10**, 10912-10920, (2016).
15 Khan, U., Kim, T. H., Ryu, H., Seung, W. & Kim, S. W. Graphene tribotronics for electronic skin and touch screen applications. *Adv. Mater.* **29**, 1603544, (2017).
16 Wang, Z. L. & Wang, A. C. On the origin of contact-electrification. *Mater. Today* **30**, 34-51, (2019).
17 Lin, S. Q., Xu, L., Tang, W., Chen, X. Y. & Wang, Z. L. Electron transfer in nano-scale contact electrification: Atmosphere effect on the surface states of dielectrics. *Nano Energy* **65**, 103956, (2019).
18 Lin, S., Xu, L., Zhu, L., Chen, X. & Wang, Z. L. Electron transfer in nanoscale contact electrification: Photon excitation effect. *Adv. Mater.* **31**, 1901418, (2019).
19 Lin, S., Xu, L., Chi Wang, A. & Wang, Z. L. Quantifying electron-transfer in liquid-solid contact electrification and the formation of electric double-layer. *Nat. Commun.* **11**, 399, (2020).
20 Kim, S. et al. Rewritable ghost floating gates by tunnelling triboelectrification for two-dimensional electronics. *Nat. Commun.* **8**, 15891, (2017).
21 Yang, H. M. et al. Physically cross-linked homopolymer ion gels for high performance electrolyte-gated transistors. *ACS Appl. Mater. Interfaces* **9**, 8813-8818, (2017).
22 Visentin, A. F. & Panzer, M. J. Poly(ethylene glycol) diacrylate-supported ionogels with consistent capacitive behavior and tunable elastic response. *ACS Appl. Mater. Interfaces* **4**, 2836-2839, (2012).
23 Tang, B. X., White, S. P., Frisbie, C. D. & Lodge, T. P. Synergistic increase in ionic conductivity and modulus of triblock copolymer ion gels. *Macromolecules* **48**, 4942-4950, (2015).
24 Shi, L. et al. Highly stretchable and transparent ionic conductor with novel

hydrophobicity and extreme-temperature tolerance. *Research* **2020**, 2505619, (2020).
25 Liu, Q., Nian, G., Yang, C., Qu, S. & Suo, Z. Bonding dissimilar polymer networks in various manufacturing processes. *Nat. Commun.* **9**, 846, (2018).
26 Le Floch, P. et al. Wearable and washable conductors for active textiles. *ACS Appl. Mater. Interfaces* **9**, 25542-25552, (2017).
27 Bai, Y. Y. et al. Transparent hydrogel with enhanced water retention capacity by introducing highly hydratable salt. *Appl. Phys. Lett.* **105**, 151903, (2014).
28 Braga, D., Gutierrez Lezama, I., Berger, H. & Morpurgo, A. F. Quantitative determination of the band gap of WS_2 with ambipolar ionic liquid-gated transistors. *Nano Lett.* **12**, 5218-5223, (2012).
29 Bisri, S. Z., Shimizu, S., Nakano, M. & Iwasa, Y. Endeavor of iontronics: From fundamentals to applications of ion-controlled electronics. *Adv. Mater.* **29**, 1607054, (2017).
30 Nie, S., He, Y. L., Liu, R., Shi, Y. & Wan, Q. Low-voltage oxide-based synaptic transistors for spiking humidity detection. *IEEE Electron Device Lett* **40**, 459-462, (2019).
31 Park, Y. D. et al. Polyelectrolyte interlayer for ultra-sensitive organic transistor humidity sensors. *ACS Appl. Mater. Interfaces* **5**, 8591-8596, (2013).
32 Zhao, D., Fabiano, S., Berggren, M. & Crispin, X. Ionic thermoelectric gating organic transistors. *Nat. Commun.* **8**, 14214, (2017).
33 Yoshida, M. et al. Gate-optimized thermoelectric power factor in ultrathin WSe_2 single crystals. *Nano Lett.* **16**, 2061-2065, (2016).
34 Lai, Q. et al. Ionic/electronic hybrid materials integrated in a synaptic transistor with signal processing and learning functions. *Adv. Mater.* **22**, 2448-2453, (2010).
35 Kim, K., Chen, C. L., Truong, Q., Shen, A. M. & Chen, Y. A carbon nanotube synapse with dynamic logic and learning. *Adv. Mater.* **25**, 1693-1698, (2013).
36 Zhu, L. Q., Wan, C. J., Guo, L. Q., Shi, Y. & Wan, Q. Artificial synapse network on inorganic proton conductor for neuromorphic systems. *Nat. Commun.* **5**, 3158, (2014).
37 Liu, Y. H., Zhu, L. Q., Feng, P., Shi, Y. & Wan, Q. Freestanding artificial synapses based on laterally proton-coupled transistors on chitosan membranes. *Adv. Mater.* **27**, 5599-5604, (2015).
38 Tian, H. et al. Graphene dynamic synapse with modulatable plasticity. *Nano Lett.* **15**, 8013-8019, (2015).
39 Xu, W., Min, S. Y., Hwang, H. & Lee, T. W. Organic core-sheath nanowire artificial synapses with femtojoule energy consumption. *Sci. Adv.* **2**, e1501326, (2016).
40 Jiang, J. et al. 2D MoS_2 neuromorphic devices for brain-like computational systems. *Small* **13**, 1700933, (2017).
41 Yang, C. S. et al. A synaptic transistor based on quasi-2D molybdenum oxide. *Adv. Mater.* **29**, 1700906, (2017).
42 Feng, P. et al. Printed neuromorphic devices based on printed carbon nanotube thin-film transistors. *Adv. Funct. Mater.* **27**, 1604447, (2017).
43 Zhu, J. et al. Ion gated synaptic transistors based on 2D van der Waals crystals with tunable diffusive dynamics. *Adv. Mater.* **30**, 1800195, (2018).
44 John, R. A. et al. Synergistic gating of electro-iono-photoactive 2D chalcogenide neuristors: Coexistence of hebbian and homeostatic synaptic metaplasticity. *Adv. Mater.* **30**, 1800220, (2018).
45 Yang, J. T. et al. Artificial synapses emulated by an electrolyte-gated tungsten-oxide transistor. *Adv. Mater.*, 1801548, (2018).
46 Kim, Y. et al. A bioinspired flexible organic artificial afferent nerve. *Science* **360**, 998-1003, (2018).
