# Supplementary material for: Contact-electrification-activated artificial afferents at femtojoule energy
Source: Nat Commun. 2021 Mar 11;12:1581. doi: 10.1038/s41467-021-21890-1 (PMC7952391; doi:10.1038/s41467-021-21890-1)
Supplement: Supplementary file 2 — Description of Additional Supplementary Files [file 41467_2021_21890_MOESM2_ESM.pdf]

Description of Additional Supplementary Files

File Name: Supplementary Movie 1
Description: Spatial tactile information recognition by the contact-electrification-activated artificial afferent.

File Name: Supplementary Movie 2
Description: Temporal tactile information recognition by the contact-electrification-activated artificial afferent.
