# Supplementary material for: Contact-electrification-activated artificial afferents at femtojoule energy
Source: Nat Commun. 2021 Mar 11;12:1581. doi: 10.1038/s41467-021-21890-1 (PMC7952391; doi:10.1038/s41467-021-21890-1)
Supplement: Supplementary file 5 — Reporting Summary [file 41467_2021_21890_MOESM5_ESM.pdf]

nature research

Corresponding author(s): Qijun Sun

Last updated by author(s): Feb 8, 2021

Reporting Summary

Nature Research wishes to improve the reproducibility of the work that we publish. This form provides structure for consistency and transparency in reporting. For further information on Nature Research policies, see our Editorial Policies and the Editorial Policy Checklist.

Statistics

For all statistical analyses, confirm that the following items are present in the figure legend, table legend, main text, or Methods section.

n/a	Confirmed	
☐	☒	The exact sample size (*n*) for each experimental group/condition, given as a discrete number and unit of measurement
☐	☒	A statement on whether measurements were taken from distinct samples or whether the same sample was measured repeatedly
☐	☒	The statistical test(s) used AND whether they are one- or two-sided<br>*Only common tests should be described solely by name; describe more complex techniques in the Methods section.*
☐	☒	A description of all covariates tested
☐	☒	A description of any assumptions or corrections, such as tests of normality and adjustment for multiple comparisons
☐	☒	A full description of the statistical parameters including central tendency (e.g. means) or other basic estimates (e.g. regression coefficient) AND variation (e.g. standard deviation) or associated estimates of uncertainty (e.g. confidence intervals)
☐	☒	For null hypothesis testing, the test statistic (e.g. *F*, *t*, *r*) with confidence intervals, effect sizes, degrees of freedom and *P* value noted<br>*Give P values as exact values whenever suitable.*
☐	☒	For Bayesian analysis, information on the choice of priors and Markov chain Monte Carlo settings
☐	☒	For hierarchical and complex designs, identification of the appropriate level for tests and full reporting of outcomes
☐	☒	Estimates of effect sizes (e.g. Cohen's *d*, Pearson's *r*), indicating how they were calculated

Our web collection on statistics for biologists contains articles on many of the points above.

Software and code

Policy information about availability of computer code

Data collection	Code availability
Data analysis	Code availability

For manuscripts utilizing custom algorithms or software that are central to the research but not yet described in published literature, software must be made available to editors and reviewers. We strongly encourage code deposition in a community repository (e.g. GitHub). See the Nature Research guidelines for submitting code & software for further information.

Data

Policy information about availability of data

All manuscripts must include a data availability statement. This statement should provide the following information, where applicable:

- Accession codes, unique identifiers, or web links for publicly available datasets
- A list of figures that have associated raw data
- A description of any restrictions on data availability

Data Availability

Field-specific reporting

Please select the one below that is the best fit for your research. If you are not sure, read the appropriate sections before making your selection.

☐ Life sciences ☐ Behavioural & social sciences ☒ Ecological, evolutionary & environmental sciences

For a reference copy of the document with all sections, see nature.com/documents/nr-reporting-summary-flat.pdf

Ecological, evolutionary & environmental sciences study design

All studies must disclose on these points even when the disclosure is negative.

Study description	We report a contact-electrification-activated artificial afferent at femtojoule energy.
Research sample	Upon the contact-electrification effect, the induced triboelectric signals activate the ion-gel-gated MoS2 postsynaptic transistor, endowing the artificial afferent with the adaptive capacity to carry out spatiotemporal recognition/sensation on external stimuli (e.g., displacements, pressures and touch patterns) with optional working modes. The decay time of the synaptic device triggered by single action pulse is in the range of sensory memory stage. The energy dissipation of the artificial afferents is significantly reduced to as low as 11.9 fJ per spike. Furthermore, the artificial afferents are demonstrated to be capable of recognizing the spatiotemporal information of touch patterns.
Sampling strategy	Over 100 samples are investigated to verify the results in the study. More than 85% of the sample sizes (or performances) are located in the average size range with no more than 10% variation, which is sufficient to demonstrate the reliable results.
Data collection	Data are measured with relevant instruments (or equipments) and processed with the software of Origin. All the authors contribute to the data collection and analysis.
Timing and spatial scale	Data is collected from Jan 2018 to Jan 2021. During the data collection and analysis, we continuously try to improve the results compared with the literatures and discussion results by all the authors. All the data is collected in Beijing Institute of Nanoenergy and Nanosystems, Chinese Academy of Sciences.
Data exclusions	The date of samples which can't work is excluded. The data which is not closely related with core of the manuscript is not included. The exclusion criteria are not pre-established.
Reproducibility	All the samples are tested for 20-50 times to verify the reproducibility. Sample to sample differences are carefully analyzed to re-check the sample preparation methods to achieve good reproducibility. All attempts to repeat the experiments were successful.
Randomization	The samples are allocated according to the fabrication process, experiment conditions and sample structures.
Blinding	All the data are measured and analyzed as it is after collection. All the data needs to be measured with instruments and analyzed based on calculations, so blinding is not relevant to this study.

Did the study involve field work? ☐ Yes ☒ No

Reporting for specific materials, systems and methods

We require information from authors about some types of materials, experimental systems and methods used in many studies. Here, indicate whether each material, system or method listed is relevant to your study. If you are not sure if a list item applies to your research, read the appropriate section before selecting a response.

Materials & experimental systems

n/a	Involved in the study
☒	☐ Antibodies
☒	☐ Eukaryotic cell lines
☒	☐ Palaeontology and archaeology
☒	☐ Animals and other organisms
☒	☐ Human research participants
☒	☐ Clinical data
☒	☐ Dual use research of concern

Methods

n/a	Involved in the study
☒	☐ ChIP-seq
☒	☐ Flow cytometry
☒	☐ MRI-based neuroimaging
